# Taxonomy of *Vincetoxicum* s.str. (Asclepiadoideae, Apocynaceae) from southern Asia including three new species and resurrected names

**DOI:** 10.3897/phytokeys.179.62514

**Published:** 2021-06-17

**Authors:** Sayed Afzal Shah, Amir Sultan, Jun Wen, Zahid Ullah, Surat Un Nisa, Zhumei Ren, Muhammad Maqsood Alam, Javed Iqbal, Abdul Samad Mumtaz

**Affiliations:** 1 Department of Plant Sciences, Quaid-i-Azam University, Nurpur Road, Islamabad 45320, Pakistan Quaid-i-Azam University Islamabad Pakistan; 2 Department of Biological Sciences, National University of Medical Sciences, The Mall, Abid Majeed Road, Rawalpindi, Punjab 46000, Pakistan National University of Medical Sciences Rawalpindi Pakistan; 3 National Herbarium, National Agriculture Research Centre (NARC), Park Road, Islamabad, Pakistan National Agriculture Research Centre Islamabad Pakistan; 4 Department of Botany, National Museum of Natural History, MRC-166, Smithsonian Institution, Washington DC 20013-7012, USA National Museum of Natural History Washington United States of America; 5 Center for Plant Sciences and Biodiversity, University of Swat, Kanju Township, Swat, Pakistan University of Swat Swat Pakistan; 6 School of Life Science, Shanxi University, Taiyuan, Shanxi, China Shanxi University Taiyuan China

**Keywords:** Afghanistan, asclepiads, endemic species, India, Pakistan, taxonomic revision, Tylophorinae, typification

## Abstract

This paper presents a taxonomic study of genus *Vincetoxicum* s.str. from southern Asia. Eleven regional endemic species are recognized on the basis of herbarium studies and fieldwork. Three new species are described: *V. lenifolium***sp. nov.** (endemic to Pakistan), *V. stewartianum***sp. nov.** (endemic to India), and *V. subcanescens***sp. nov.** (endemic to Pakistan, Kashmir and Tibet). Three species names, *V. cabulicum*, *V. glaucum* and *V. kenouriense*, previously treated as synonyms of *V. glaucum*, *V. canescens* and *V. hirundinaria*, respectively, are resurrected. A neotype is designated for the Afghani endemic *V. cabulicum*. A lectotype is chosen from the syntypes of *V. glaucum*. We resolve the long-standing taxonomic problems in three species complexes: *V. arnottianum*, *V. luridum*, *V. sakesarense*, and *V. stocksii*; *V. glaucum*, *V. canescens* and *V. cabulicum*; and *V. hirundinaria* and *V. kenouriense*. Geo-taxonomic distinctions of southern Asian taxa are highlighted by excluding from henceforth the long misrecognized western Eurasian taxa *V. canescens* and *V. hirundinaria*. Furthermore, a detailed account of the genus including illustrations of whole plants, leaves and corona, distribution maps, a taxonomic key, morphological descriptions, synonymy, notes, and information on phenology, distribution and habitats is provided. Finally, provisional conservation assessments are provided, which indicate that *V. cardiostephanum* and *V. sakesarense* are critically endangered.

## Introduction

The subtribe Tylophorinae K. Schum. of Asclepiadoideae, Apocynaceae, comprises the genera *Pentatropis* R.Br. ex Wight & Arnott with five species, and *Vincetoxicum* Wolf with approximately 200 species. Liede-Schumann et al. (2012) expanded *Vincetoxicum* to include *Biondia* Schlechter, *Blyttia* Arnott, *Diplostigma* K. Schumann, *Goydera* Liede, *Pleurostelma* Baillon, *Rhyncharrhena* Mueller, and *Tylophora* R. Brown. The resulting new combinations, new names and typifications were recently published in Liede-Schumann and Meve (2018). Members of *Vincetoxicum* are distributed in Africa, Arabia, Australia and Eurasia. The plants are either erect undershrubs or twiners, often with transparent latex, leaves are simple and opposite, and flowers are mostly small with staminal corona lobes and round pollinia. The infra-generic taxa are often difficult to identify. The most important characters for identification are usually confined to the small (± 5 mm long) flowers. The key floral characters include color, dimensions, and internal indumentum of the corolla lobes, and shape, orientation and size of the corona lobes. The vegetative morphological features are often strikingly homomorphic among closely related species.

From the taxonomic viewpoint, *Vincetoxicum* is commonly considered a difficult genus, and has been confused by several authors with *Cynanchum* (e.g. Wight 1834; Hooker 1883; Parker 1956; Kitamura 1960; Li et al. 1995), a view opposed by others (e.g. Decaisne 1844; Boissier 1879; Bullock 1958; Rechinger 1970; Markgraf et al.1972; Browicz 1978; Liede 1996). The genus has gained attention during the past two decades along with its allies in several independent studies (e.g., Liede and Kunze 2002; Yamashiro et al. 2004a; Liede-Schumann et al. 2016; Liede-Schumann et al. 2012), leading to a new generic circumscription within Tylophorinae. Infra-generic taxonomy, however, has earned meager attention. New species discoveries from different parts of Eurasia over the past four decades (e.g. Ali and Khatoon 1982; Zaeifi 1999; Yamashiro et al. 2004b; Kidyoo 2016; Jiang et al. 2018; Shah et al. 2018) indicate that *Vincetoxicum* is a complex genus and that regional taxonomic revisions are important to uncover the patterns of species diversity. The need for regional taxonomic revisions is also emphasized by Liede-Schumann et al. (2012, 2016). Molecular phylogenetic studies are also fundamentally important to clarify the boundaries between cryptic species and different geographic groups.

Southern Asian members of *Vincetoxicum* s.str. have been treated periodically in different floristic and taxonomic accounts (e.g. Wight 1834; Decaisne 1844; Wight 1850; Boissier 1879; Hooker 1883; Rechinger 1970). Wight (1834) was the first on *Vincetoxicum* to describe fifteen *Cynanchum* species from British India including *Cynanchum arnottianum* Wight, *C. glaucum* Wight and *C. kenouriense* Wight. A decade later, Decaisne (1844) moved these three species to *Vincetoxicum* whereas *C. glaucum* was synonymized with *V. canescens* (Willd.) Decaisne, a treatment which Boissier (1879) also adopted. Hooker (1883) reinstated *C. glaucum* but synonymized *V. kenouriense* (Wight) Wight with *C. vincetoxicum* (L.) Pers., a synonym of *V. hirundinaria* Medikus. Rechinger (1970) described the new species *V. cardiostephanum* (Rech. f.) Rech. f., from Afghanistan and recognized *V. glaucum* in a broader sense, merging another Afghani species, *V. cabulicum* Bornm., with the former. Hara et al. (1982) recognized *V. hirundinaria* in a much broader sense, including one additional subspecies V. hirundinaria subsp. glaucum (Wall. ex Wight) H. Hara with which Rechinger’s (1970)*V. glaucum* was also synonymized.

The revision of Pakistani *Vincetoxicum* (Ali and Khatoon 1982) was the first detailed study on this group from southern Asia. The authors recognized six species from Pakistan including *V. hirundinaria* and *V. canescens*. These two species were recognized in a broader sense, merging Rechinger’s *V. glaucum* into the latter. According to Ali and Khatoon (1982), these two species have a wide range from southern Asia westwards to the Mediterranean region (*V. canescens*) and Europe (*V. hirundinaria*). In a phylogenetic analysis, Liede-Schumann et al. (2016) recently recognized the Pakistani specimens as *V. glaucum* and distinguished them from *V. canescens*. The former came out as a member of the Western Himalayan subclade while *V. canescens* was grouped in a small Mediterranean subclade with *V. creticum* Browicz and *V. tmoleum* Boissier. The phylogenetic analysis of Liede-Schumann et al. (2016) disintegrated the *V. canescens* complex into the Mediterranean (*V. canescens*) and western Himalayan (*V. glaucum*) entities. Despite these advancements, to solve the *V. glaucum* complex, which includes plants of the so-called *V. cabulicum* from Afghanistan and *V. glaucum* from Pakistan, Kashmir, Tibet (China), India and Nepal, an inclusive taxonomic analysis is needed. Similarly, *V. hirundinaria* has traditionally been regarded as a variable species and spans a wide range in European Russia, Western Siberia, Turkey, the Caucasus, Kashmir, Pakistan (Hazara, Waziristan) and the Himalayas up to Sikkim (Ali 1983). Southern Asian accessions of *V. hirundinaria* were lacking in Liede-Schumann et al. (2016). Therefore, a comparison of accessions of *V. hirundinaria* throughout its range is necessary. More recently, Shah et al. (2018) elucidated the long-misunderstood complex of closely related, purple-flowered species *V. arnottianum*, *V. sakesarense* Ali & Khatoon and *V. stocksii* Ali & Khatoon, which resulted in the addition of a new species *V. luridum* Stocks ex S.A. Shah that is endemic to Balochistan (Pakistan).

This research revises *Vincetoxicum* s.str. from southern Asia. Prior to this study, a total of seven *Vincetoxicum* s.str. species were recognized in southern Asia viz., *V. arnottianum* (Pakistan, Kashmir and India), *V. cardiostephanum* (Afghanistan and Kurram valley of Pakistan), *V. glaucum* (Nepal westwards to Afghanistan), *V. hirundinaria* (Nepal westwards to Pakistan), *V. luridum* (endemic to Pakistan), *V. sakesarense* (endemic to Pakistan) and *V. stocksii* (endemic to Pakistan). In comparison to previous studies, we recognize 11 *Vincetoxicum* s.str. species in southern Asia (Table 1). We describe three new species and modify previously existing descriptions. *Vincetoxicum luridum* was published just recently by Shah et al. (2018), so its description is not repeated in this paper. All species recognized in this treatment are endemic to southern Asia. The long misapplied species names *V. canescens* and *V. hirundinaria* are hereby excluded from southern Asia which breaks the connections between the southern Asian and the rest of the Eurasian, especially the western Eurasian, *Vincetoxicum* species.

**Table 1.** Provisional conservation assessment and areas of endemism of southern Asian *Vincetoxicum*.

Species	AOO (km^2^)	EOO (km^2^)	Provisional conservation assessment (IUCN 2017)	Endemic to
*Vincetoxicum arnottianum*	88	30,680	NT	Pakistan, India
*V. cabulicum*	20	11,583	VU	Afghanistan
*V. cardiostephanum*	12	53	CR	Afghanistan, Pakistan
*V. glaucum*	20	16,215	DD	India, Nepal
*V. kenouriense*	116	263,512	LC	Pakistan, India, Nepal, Bhutan
*V. lenifolium*	16	836	EN	Pakistan
*V. luridum*	52	28,013	NT	Pakistan
*V. sakesarense*	8	–	CR	Pakistan
*V. stewartianum*	4	–	DD	India
*V. stocksii*	36	6,123	VU	Pakistan
*V. subcanescens*	76	67,173	LC	Pakistan, India, Tibet (China)

## Materials and methods

**Figure 1.**
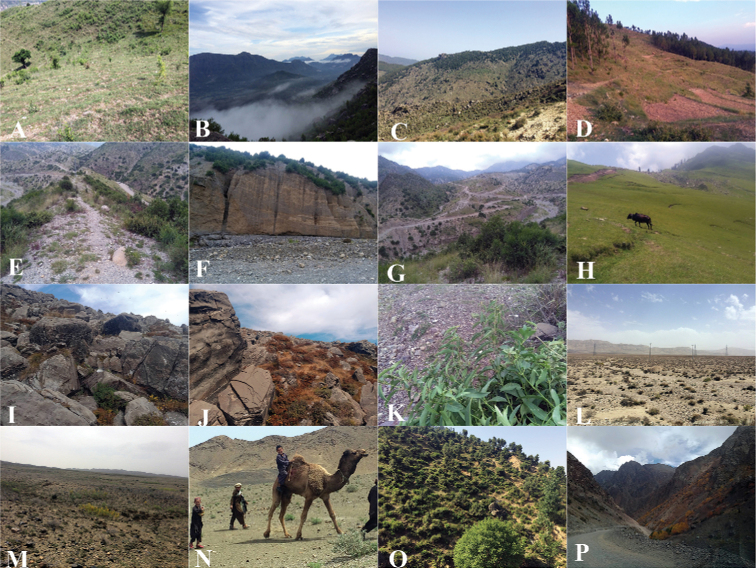
Habitats of *Vincetoxicum* species **A–D***V. arnottianum***E–G***V. cardiostephanum***H–J***V. kenouriense***K***V. lenifolium***L***V. luridum***M, N***V. stocksii***O, P***V. subcanescens*.

We morphologically examined approximately 800 herbarium specimens, including our recent collections (2015–17) from Pakistan. We conducted a total of 65 field visits to cover Himalaya, Hindukush, and Sulaiman, representing the major mountain ranges of Pakistan (Fig. 1) and extending into Afghanistan and India. We collected specimens in flowering and fruiting stages for improved characterization. We conserved live plants of *V. arnottianum*, *V. cardiostephanum*, *V. kenouriense*, *V. lenifolium*, *V. luridum*, *V. sakesarense*, *V. stocksii* and *V. subcanescens* at the Botanical Conservatory, National Agriculture Research Centre (**NARC**); Islamabad, Pakistan, for morphological observations and future research. We deposited our fresh collections including the type specimens in RAW and US. We used the live plants for determination of shape and dimensions of smaller structures including corona, gynostegium and calycine colleters. Colors of corollas, fruits, etc., are described from live material or herbarium labels. We carried out morphological examinations at HUP, ISL, KUH, PPFI, PMNH, RAW, and US and received herbarium loans from B, BM, GH, GOET, K, MO, and NY at US. We also retrieved digitized herbarium specimens from the databases JSTOR Global Plants (https://plants.jstor.org/), E, and P and used either the website’s measurement system or IMAGEJ for the downloaded images. Herbarium acronyms follow Thiers (2020). We studied the type specimens of the previously known names *V. arnottianum*, *V. canescens*, *V. hirundinaria*, *V. kenouriense*, *V. sakesarense*, *V. stocksii*, and *V. glaucum*. The type specimen of *V. cabulicum* was destroyed in B during World War II, therefore, we studied other specimens from the same area and designated a neotype. We followed the terminology of Ali and Khatoon (1982) for floral characters, and Harris and Harris (1994) for general morphological characters. For illustrations of the whole plant, we mostly used fresh plants and sometimes herbarium specimens. We also illustrated the leaf outlines mainly from fresh plants. During illustrations of corona types, we ensured the best angles that help determine the accurate shape of corona lobes and their orientation. We assessed the conservation status following the IUCN Red List Categories and Criteria (IUCN 2019) using the GIS-based method of Moat (2007) as implemented in the online assessment tool GeoCat (http://geocat.kew.org). The Extent of Occurrence (**EOO**) measures the range of the species, and the Area of Occupancy (**AOO**) represents the number of occupied points within that range based on the default grid size of 2 km^2^.

## General description of southern Asian *Vincetoxicum* s.str.

### Description of vegetative parts

South Asian *Vincetoxicum* s.str. species are mostly found in mountains predominantly at higher elevations ranging from 750 to 3650 m. The plants are perennial, growing one to several stems from the rhizomatous base in spring and becoming dormant in winter. They lack milky latex and have clear latex instead. Stems are mostly longitudinally striate and pubescent all around or along one to two lines. Multiple patterns of pubescence are often found in the same species. Pubescence may be dense or sparse depending on the species. Trichomes are exclusively uniseriate, 3–5 celled, falcate or straight, with the terminal cell sometimes inflated. The phyllotaxis is opposite and decussate. However, some plants exhibit leaves in whorls or pseudo-whorls of 3–6 leaves. Laminae are discolorous and of different shapes ranging from linear to broadly ovate. Margins are always smooth. The shape of the apex and base vary depending upon the species. Laminae of the purple-flowered *Vincetoxicum* species are conduplicate. The adaxial veins, especially the midrib, are pubescent in most taxa. A cluster of small glandular structures, called colleters, are found at the bases of the laminae on the adaxial surface. Depending on the species, the remainder of foliar veins and surfaces are either glabrous or variously hairy. Foliar trichomes resemble those on the stems. Flowers are typically clustered in dense pseudo-umbels either sessile or sub-sessile or both. The pubescence on peduncles is identical to that of the stem in density, but that of the pedicels is often different.

### Description of reproductive parts

The sepals are fused basally into a short calyx tube and the calyx lobes are usually adpressed to the corolla. The corolla forms a short, usually glabrous tube. The corolla lobes are either green, whitish green, purple or purple in the lower half and green up to the apex. They are either free from each other to the apex or twisted clockwise up to the apices that enclose the internal parts (in *V. arnottianum* and *V. sakesarense* only). Inside, the corolla lobes are either variously pubescent or glabrous; rarely, the trichomes are caducous. The corona is composed of five lobes basally attached to the stamens, hence called staminal corona. The corona is mostly prominent and separate from the stamens except at the base. It is the most important structure from a taxonomic point of view. However, it is difficult to study because of its small size, usually less than one millimeter in dimension. Mostly, it is fleshy and loses water content during herbarium pressing, which somewhat alters the shape and size of the lobes. Studying dried as well as fresh and/or rehydrated flowers is the most precise way of determining the shape and size of corona lobes. Another important character is the length ratio of gynostegium and corona lobes. The gynostegium possesses pollinaria in the anther locules. Each locule possesses one pollinium. The orientation of pollinaria should be noted during dissection of flowers.

The fruit is a typical asclepiad follicle. The two ovaries, if successfully pollinated, develop into paired follicles. The follicles are fused at the base, but diverge at various degrees. Each follicle contains 10–25 seeds attached to a central axis. Seeds are dispersed by wind with the help of a coma, i.e. a tuft of approximately 2–3 cm long white hairs attached to the seed apex. Margins of seeds (wings) are thin all around except at the apex.

## Taxonomic treatment

### Southern Asian *Vincetoxicum* s.str.

Rhizomatous perennial undershrubs, sometimes herbaceous, up to 1 m tall, latex clear. Stems erect, round, longitudinally striate, glabrous or pubescent all-around with simple uniseriate trichomes or along 1–2 longitudinal lines, internodes 1–13 cm long. Leaves simple, mostly opposite, rarely both opposite and 3–4-whorled (only in *V. arnottianum*), decussate, lower and upper leaves smaller than middle ones, spreading or rarely pendent (only in *V. cardiostephanum*), petiolate or rarely sub-sessile (only in *V. hirundinaria* and *V. cabulicum*); petioles 1–18 mm long, mostly pubescent along the adaxial channel or all around or glabrous; lamina variously shaped, mostly narrowly ovate to broadly ovate, 1.5–13 × 1–6.5 cm; apex acute to shortly acuminate; margins simple; secondary veins opposite or sub-opposite, 8–14 on each side of midrib, eucamptodromous, normally conspicuous, sometimes inconspicuous, rarely highly prominent (only in *V. kenouriense*); leaf surface glabrous or with different indumentum types of uniseriate simple trichomes: sub-glabrous, pubescent, densely pubescent, sub-canescent. Inflorescences lax to dense-flowered pseudo-umbels, mostly sessile, sometimes pedunculate, rarely long-pedunculate; peduncles glabrous or pubescent. Flowers pentamerous except carpels, pedicellate; calyx green, persistent in fruiting, polysepalous except base, lobes 1–2 mm long, margins thin, abaxial surface pubescent or glabrous, calycine colleters single or in pairs, rarely in threes; corolla lobes ovate to oblong to rarely oblong-linear (in *V. arnottianum*), 2–5 × 1–2.5 mm, straight or twisted clockwise, variously coloured: mostly purple, but also green, bicolored (only in Balochistani species) or yellowish, internally glabrous or pubescent or bearded; corolla tube rarely pubescent inside (only in *V. cardiostephanum*); gynostegial corona lobes variously shaped: clavate, deltoid, deltoid-rhomboid, subulate, oblong, obovate, ovate, rhomboid, triangular, shorter or longer than or equal in length to the gynostegium, apices of corona lobes round or acute or fimbriate (only in *V. glaucum*), convergent or divergent, bases connected by soft basal tissues; pollinaria pendent, corpusculum mostly loosely embedded in the five apical corners of the gynostegium (but deeply embedded in *V. glaucum*), caudicles mostly ascending from corpuscular to pollinarium end, rarely slender along the entire length (only in *V. luridum*), whitish, pollinia yellow. Stamens with thin apical anther appendages covering the gynostegium apex and pollinia. Follicles narrowly fusiform with mostly acuminate apex, 4–9.5 × 0.4–13 cm, inconspicuously striate, mostly glabrous at maturation, sometimes sparsely pubescent. Seeds mostly ovate, 4–10 × 2.2–7.5 mm, brown, winged on all sides except at apex, wing up to 1 mm wide, apex comose; coma 2–3 cm long, white.

### A taxonomic key to southern Asian *Vincetoxicum*

**Table:** 

1	Leaves strongly discolorous, tertiary and quaternary veins prominent; flowers green, corona lobes triangular	***V. kenouriense***
–	Leaves weakly discolorous, tertiary and quaternary veins inconspicuous; flowers green or purple or bicolored, corona lobes not triangular	**2**
2	Flowers and leaves pendent, corolla tube pubescent internally	***V. cardiostephanum***
–	Flowers and leaves not pendent, corolla tube glabrous internally	**3**
3	Corolla lobes glabrous within	***V. lenifolium***
–	Corolla lobes hairy within	**4**
4	Corona lobes exceeding gynostegium in length	**5**
–	Corona lobes not exceeding gynostegium in length	**8**
5	Apices of corona lobes convergent; leaves hairy on both sides	**6**
–	Apices of corona lobes divergent; leaves not hairy on both sides	**7**
6	Plants densely pubescent; corona lobes ovate; follicles up to 6 cm long	***V. luridum***
–	Plants sparsely pubescent; corona lobes subulate; follicles up to 9.5 cm long	***V. stocksii***
7	Leaves narrowly to broadly ovate; corona lobes longer than broad; toothed	***V. glaucum***
–	Leaves lanceolate-ovate to elliptic-ovate; corona lobes rhomboid, broader than long	***V. stewartianum***
8	Leaves narrowly to broadly ovate, sub-canescent; corona lobes obovate	**9**
–	Leaves narrowly to mostly lanceolate-ovate; corona lobes not obovate	**10**
9	Leaves subsessile, corolla and inner floral parts purple	***V. cabulicum***
–	Leaves petiolate, corolla and inner floral parts green	***V. subcanescens***
10	Corona lobes rhomboid-deltoid	***V. arnottianum***
–	Corona lobes deltoid	***V. sakesarense***

#### 1. Vincetoxicum arnottianum

Taxon classificationPlantaeGentianalesApocynaceae

(Wight) Wight, Icones Pl. Ind. Or. t. 1614. 1850.

1F589DD5-A969-5372-8199-B77C0857EAD8

**Figure 2.**
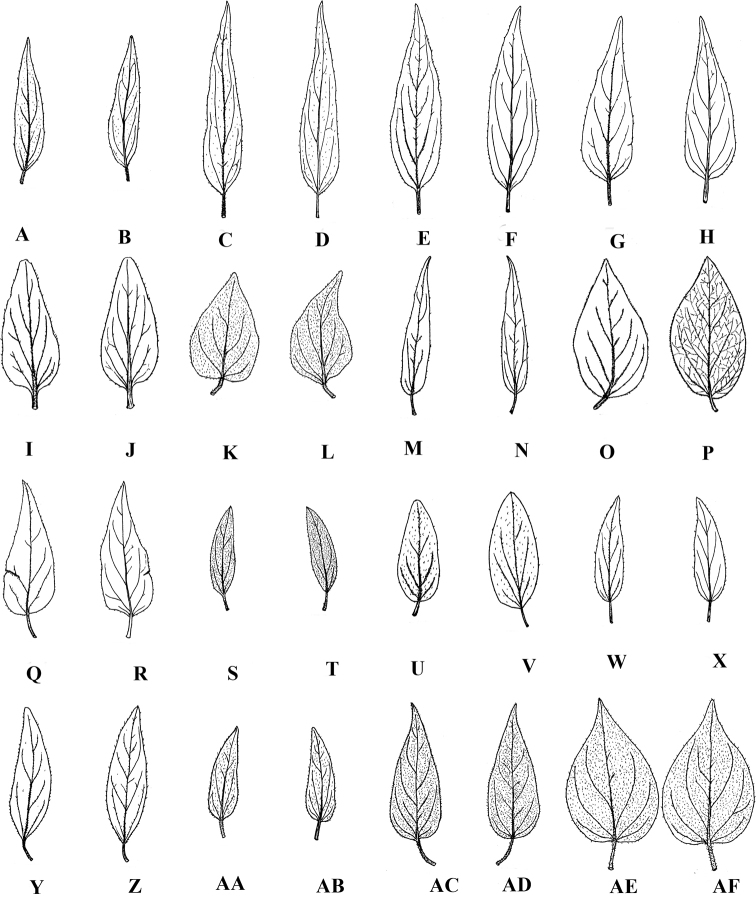
Outlines of leaves of southern Asian *Vincetoxicum*. All illustrated specimens are housed at RAW. The first image in each pair corresponds to the adaxial surface and the second to the abaxial surface. All leaves have been taken from the middle of the stem **A, B**Vincetoxicum sp. aff. arnottianum from *S.A. Shah SAS-9***C, D**Vincetoxicum sp. aff. arnottianum from *S.A. Shah SAS-46***E, F**Vincetoxicum sp. aff. arnottianum from *R. Khan SAS-41***G, H***V. arnottianum* from *S.A. Shah & F. Rahman SAS-3***I, J***V. arnottianum* from *S.A. Shah & B. Ali SAS-4***K, L***V. cabulicum* from *D. Podlech 15814***M, N***V. cardiostephanum* from *S.A. Shah, M. Turi et al. SAS-40***O, P***V. kenouriense* from *S.A. Shah & L. Ahmad SAS-37***Q, R***V. lenifolium* from *S.A. Shah SAS-44***S, T***V. luridum* from *S.A. Shah & I. Ahmad SAS-23***U, V***V. lenifolium* from *N. Ali 1029***W, X***V. sakesarense* from *I. Ahmad 27821***Y, Z***V. stewartianum* from *R.R. Stewart 16709***AA, AB***V. stocksii* from *B. Gul, N. Khan et al. SAS-21***AC, AD***V. subcanescens* from *A. Sultan, Z. Saqib et al. SAS-52***AE, AF***V. subcanescens* from *S.A. Shah & A. Ullah SAS-8.* Drawing by M. Saleem and S.A. Shah.

**Figure 3.**
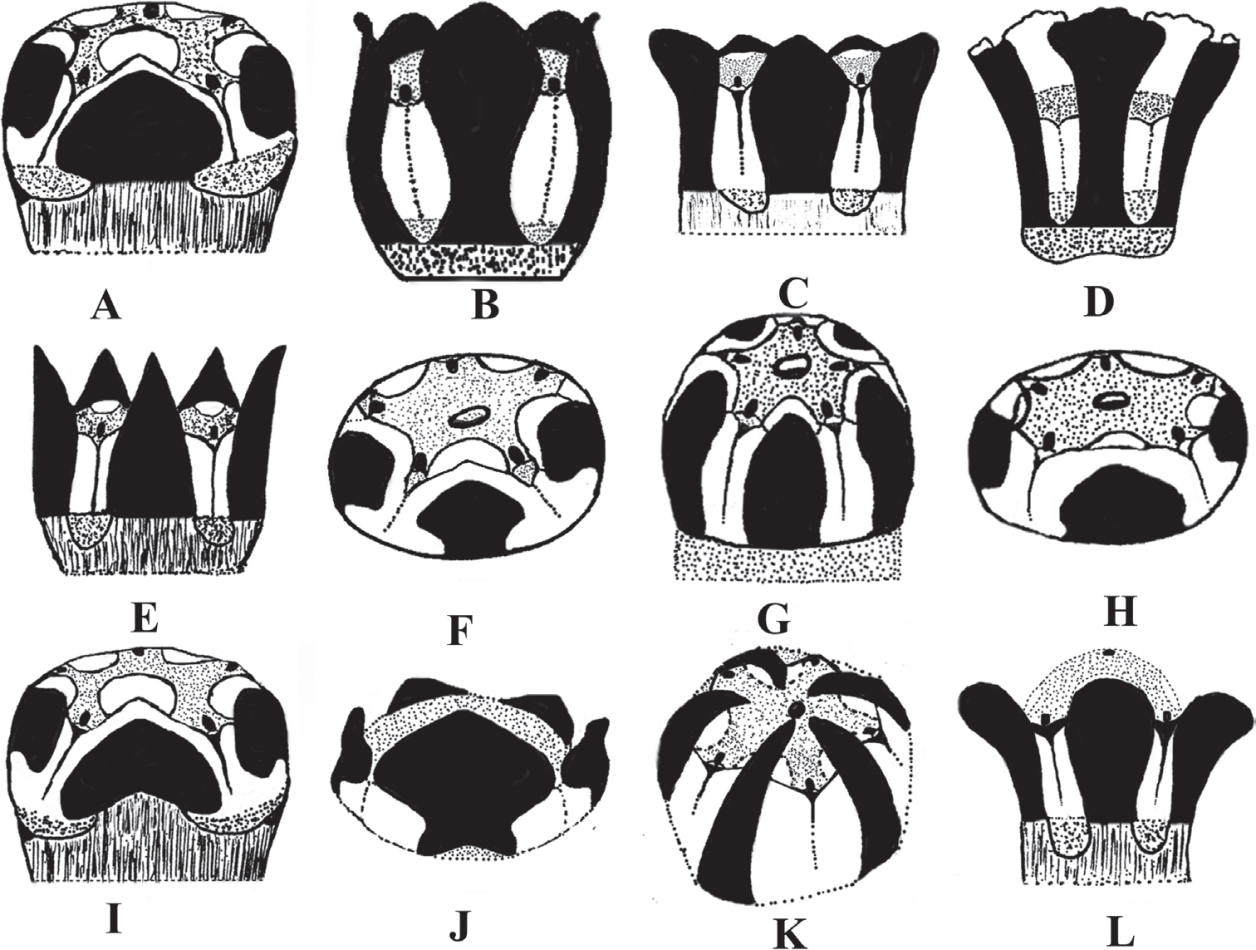
Illustration of corona types observed in southern Asian *Vincetoxicum*. The dark coloured shapes show corona lobes **A***Vincetoxicum arnottianum* from *S.A. Shah & B. Ali SAS-4* (RAW) **B***V. cabulicum* from *W. Koelz 11764* (US) **C***V. cardiostephanum* from *S.A. Shah, M. Turi et al. SAS-40* (RAW) **D***V. glaucum* from *W. Dudeeon & L. A. Kenoyer 56* (MO) **E***V. kenouriense* from *S.A. Shah & L. Ahmad SAS-37* (RAW) **F***V. lenifolium* from *S.A. Shah SAS-44* (RAW) **G***V. luridum* from *S.A. Shah & I. Ahmad SAS-23* (RAW) **H** A variable specimen of *V. lenifolium* from *N. Ali 1029* (RAW) **I***V. sakesarense* from *I. Ahmad 27821* (RAW) **J***V. stewartianum* from *R.R. Stewart 16709* (RAW) **K***V. stocksii from J.F. Duthie 18918* (RAW) **L***V. subcanescens* from *S.A. Shah & A. Ullah SAS-8* (RAW). Drawing by M. Saleem and S.A. Shah.

**Figure 4.**
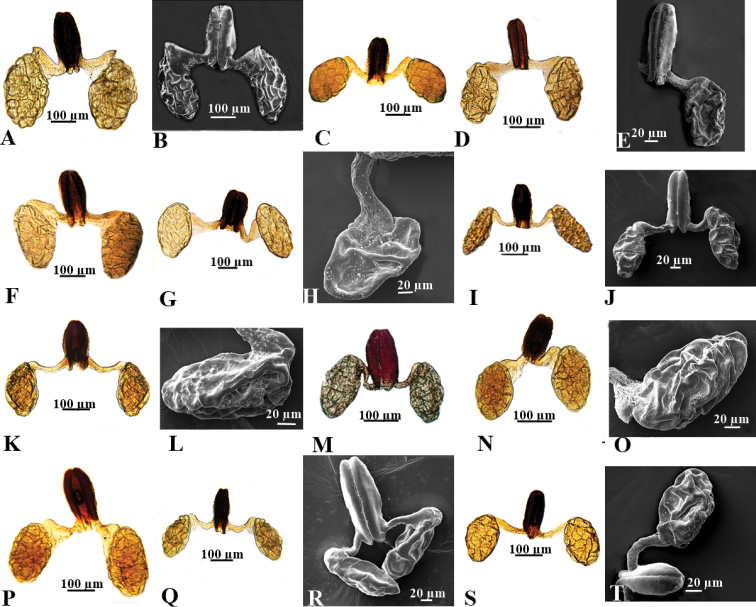
Pollinaria of southern Asian *Vincetoxicum***A, B***V. arnottianum***C***V. cabulicum***D, E***V. cardiostephanum***F***V. glaucum***G, H***V. kenouriense***I, J***V. lenifolium***K, L***V. luridum***M***V. lenifolium***N, O***V. sakesarense***P***V. stewartianum***Q, R***V. stocksii***S, T***V. subcanescens*.

**Figure 5.**
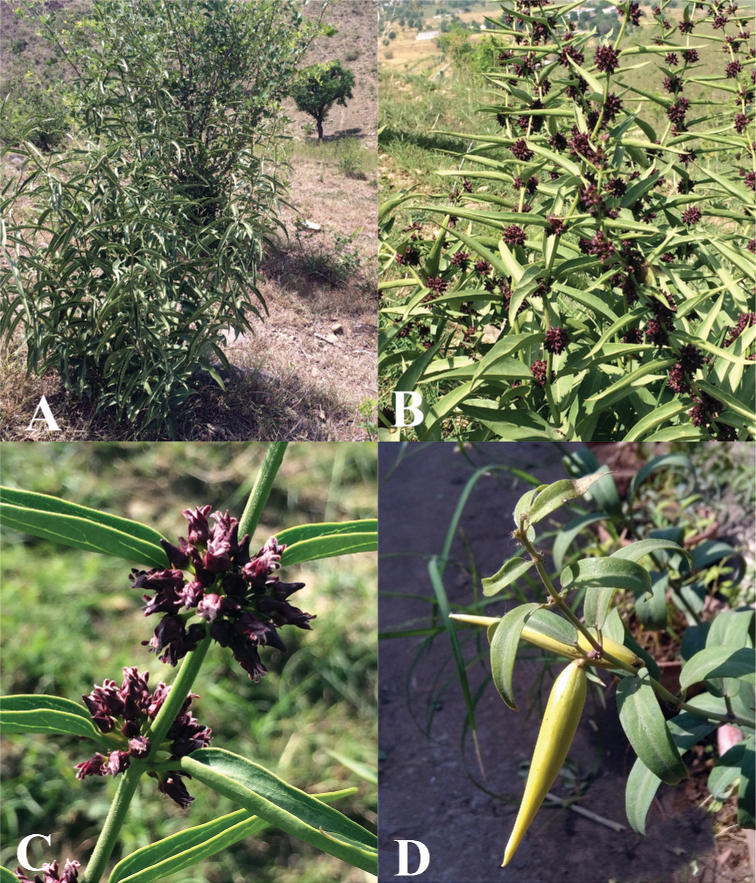
Field photographs of *Vincetoxicum arnottianum***A** habit **B** leaves and inflorescences **C** flowers **D** follicles. Photos by S.A. Shah.

**Figure 6.**
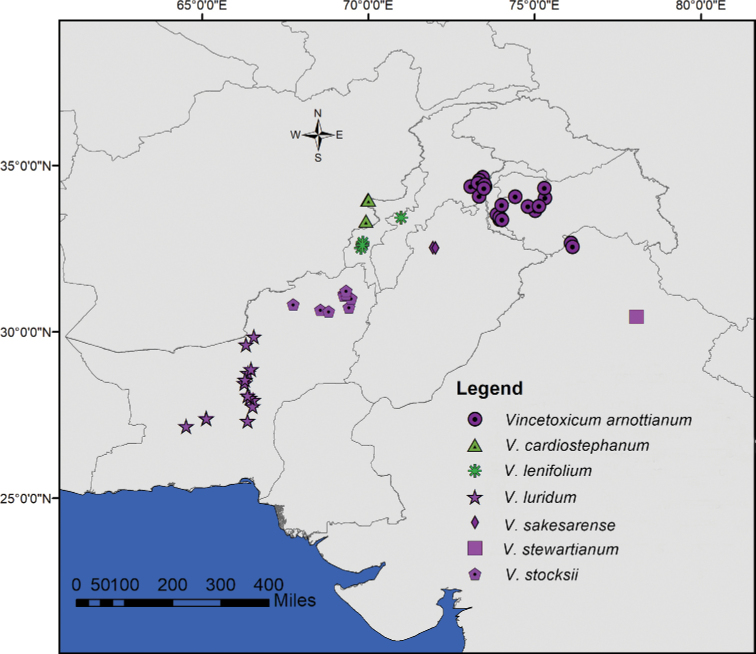
Geographic distribution of southern Asian *Vincetoxicum*. The colour of the symbols corresponds to the colour of the corolla.

Figs 2G–J , 3A , 4A, B , 5 , 6

##### Type.

India. Kashmir: ‘In itinere ad Cashmere’, *Royle, 21* (***Holotype***: K! [K000872738]).

Undershrubs, up to 100 cm tall. Stem striate, pubescent along one narrow line throughout the stem or alternating along two lines, sometimes both single and double lines found, internodes 0.5–10 cm long. Leaves usually opposite, rarely 3–4 whorled; petioles 2–13 mm long, pubescent along adaxial channel, abaxially glabrescent, rarely pubescent all around; lamina discolorous, narrowly ovate to lanceolate-ovate, 3–13 × 1–3.5 (–5) cm; apex acute to narrowly acute; base obtuse; veins visible on both sides, sometimes inconspicuous, secondary veins 8–10 (–14) on each side of midvein; both surfaces and abaxial veins glabrous, adaxial veins pubescent; margins sparsely pubescent. Inflorescences sessile, very rarely pedunculate; peduncles up to 1.5 cm long, pubescent; bracts linear with ciliate margins, ±1 mm long; pedicels 1–5 mm long, pubescent along single or double lines, sometimes glabrous. Flowers 4.5–5.5 × 2–3 mm; sepals gradually tapering to narrowly acute or acuminate apices, up to 2 mm long, margins ciliate, sometimes pubescent on abaxial surface; calycine colleters 5 or 10, unequal in length when paired; corolla dark purple, corolla tube ca. 1 mm long, lobes twisted clockwise, bearded over the whole surface within except for the lateral margin, oblong, gradually tapering to the apex, 2.5–4 × 1–2 mm; corona deltoid-rhomboid, broader than long, 0.6–0.8 × 0.8–1 mm, reaching the bases of the staminal appendages in length or rarely equalling the gynostegium, divergent. Follicles fusiform, 4–7.5 × 0.8–1 cm, apex acuminate, surface glabrous, striations inconspicuous. Seeds reddish brown, 6–8 × 3–4.5 mm, wings up to 1 mm broad, brown dots rarely distantly present; coma up to 2 cm long.

##### Distribution and habitat.

Based on the number of past collections and our field observations, this is the most commonly occurring species of *Vincetoxicum* in southern Asia. It is strictly western Himalayan in distribution and found in India (Himachal Pradesh), Kashmir and Pakistan (Azad Jammu, Kashmir and Hazara Division). It is a deep rooted plant with a thick root stock found on open, sunny mountain slopes in association with grasses or other herbaceous flora. Its elevation ranges from 750 to 2800 m.

##### Phenology.

Flowering from April to September and fruiting from May to October.

##### Provisional conservation status.

Near threatened (Table 1). *Vincetoxicum arnottianum* is found in highly clumped and distant populations. Its populations have been mostly found in areas less than 100 m^2^ in size and consist of less than 100 plants.

##### Notes.

*Vincetoxicum arnottianum* has been confused with closely related entities for a long time. However, in recent studies (Ali and Khatoon 1982; Shah et al. 2018), those entities are regarded as separate species, namely *V. lenifolium* (this paper), *V. luridum*, *V. sakesarense* and *V. stocksii*. The inter-species relationships within the *V. arnottianum* complex (except *V. lenifolium*) were discussed in Shah et al. (2018). However, there are two associated problems that still need more clarification. Firstly, the type specimen of *V. arnottianum* was collected from western Himalaya, but the drawings on the herbarium sheet depict characters of a Balochistani species *V. luridum*, that we recently introduced as a new species in Shah et al. (2018). We hereby present our detailed observations to clarify this first problem.

Robert Wight provided two descriptions of *V. arnottianum*, notably in the protologue (Wight 1834), and a revised description (Wight 1850). In the former, he described *V. arnottianum* as a glabrous plant with oblong, obtuse or emarginate leaves, sessile umbels, 5-fid internally hairy corolla lobes, 5-fid corona lobes (shape not provided) equal to the gynostegium. The type designated was “In itinere ad Cashmere, Royle (K [K000872738])”. The locality mentioned means “on the way to Kashmir”. Kashmir (India) is part of the western Himalaya. For the revised description, Wight acquired specimens from Dr. Stocks collected from Balochistan and named them *V. luridum*. The name “*Vincetoxicum luridum*” was limited to those specimens and Stocks did not publish the species. Three specimens of that collection are housed in K, with K001235295 chosen as holotype of *V. luridum* (Shah et al. 2018). Wight (1850) regarded both K000872738 and Stocks’ collections as one species as opposed to Stocks’ view. Therefore, in the revised description, Wight (1850) added the following characters: suffruticose, climbing, branches terete, leaves succulent, umbels subsessile, many-flowered, stigma apiculate. In the geographic distribution, he mentions “Beluchistan” (correct spelling is Balochistan or Baluchistan). According to our observations, these revised characters predominantly belong to the Balochistani collections. Therefore, Wight’s (1850) revised description is based on two different taxonomic entities. Balochistan is located in south-western Pakistan, not in the Himalayas, and has its own mountain ranges far away from Kashmir (see distribution map in Shah et al. (2018). Wight (1850, p. 17) acknowledged Dr. Stocks for his collections and stated: “*This species was first taken up from rather imperfect specimens, whence some alterations have here been found necessary to adapt the character to the species. I am indebted to Dr. Stocks for the specimens from which the drawing and revised character were taken*”. On the other hand, one of the Stocks’ specimens (K001235295) has a manuscript note in Stocks’ handwriting “*figured by Wight in his Icones from my specimens as his Vincetoxicum arnottianum I doubt*”. Wight used the term “climbing” in the revised description because he observed Stocks’ specimens. He did not use this term in the 1834 description. The illustration provided in 1850 depicts a somewhat climbing habit. The type of *V. arnottianum* K000872738 does not show a climbing habit. The term “suffruticose” and other characters of the revised description, and some floral and pollinarium drawings were provided on a label of the type specimen K000872738. These observations further indicate that the revised characters as well as the drawings including the pollinarium pertain to Stocks’ specimens (Balochistan). The illustration provided in Wight (1850) was also drawn from Stocks’ specimens and perfectly resembles the Balochistani collections, not the western Himalayan specimens.

During this study, we thoroughly examined the type specimen of *V. arnottianum*, K000872738, the types of *V. luridum* (holotype K [K001235295], isotypes: K [K001235294], [K001235296]) as well as almost all *Vincetoxicum* collections from western Himalaya and Balochistan. The type of *V. arnottianum* resembled western Himalayan specimens in its characters and differed from those of Balochistan. Furthermore, the specimens of western Himalaya do not match those of Balochistan in morphological characters and the two areas are geographically distant and climatically different. These findings led us to our decision in Shah et al. (2018) to introduce *V. luridum* as a new species and maintain K000872738 as the type of *V. arnottianum*. Our detailed morphological comparison is provided hereby.

1. The type K000872738 is glabrous, which is a character of V. arnottianum (western Himalayan) as also mentioned in the protologue by Wight (1834). Vincetoxicum luridum is densely hairy.
2. In K000872738, the inner surfaces of the corolla lobes are bearded, not pilose like in V. luridum.
3. In K000872738, the inflorescences are not many-flowered like those of V. luridum.
4. In K000872738, the corolla is dark purple as mentioned in the protologue by Wight (1834). In V. luridum, the corolla lobes are bicolored (lower half purple + upper half green). In this taxon, as well as in V. stocksii, flower color can only be correctly determined in fresh flowers.
5. In K000872738, the corolla lobes do not seem twisted because the flowers are immature and pressed. However, we have observed twisted corolla lobes in V. arnottianum while studying fresh flowers. In K000872738, the apex of the flowering bud is acute rather than obtuse as in V. luridum. A twisted corolla is never found in the Balochistani specimens belonging to V. luridum.

Secondly, we have observed significant morphological variation in certain populations of *V. arnottianum*. In these populations, plants are comparatively short (ca. 1 feet or rarely up to 2 feet), leaves are either pubescent (Fig. 2A–D) or glabrous on both sides (Fig. 2E–H), flower colour either purple or rarely whitish green, corolla length varies from 2 to 5 mm, corona shape is either rhomboid or deltoid-rhomboid or rarely deltoid. The geography of these populations is different from the typical *V. arnottianum*. They occur in Rawalpindi district (Punjab province, Pakistan) and in Malakand division (Khyber Pakhtunkhwa province, Pakistan) comprising districts Buner, Chitral, Upper Dir, Lower Dir, Malakand, Shangla and Swat. Geographically, Malakand Division represents the eastern Hindukush mountain range and borders Afghanistan in the west. The major geographic disjunction between Malakand division and the range of typical *V. arnottianum* (India: Himachal Pradesh, Kashmir; and Pakistan: Azad Jammu, Kashmir, and Hazara Division) is the River Indus. Therefore, in this study, we just highlight this problem and propose to identify the plants possessing the above-mentioned variations as Vincetoxicum sp. aff. arnottianum. We also recommend further detailed studies preferably including a molecular analysis using variable markers to determine whether these populations could potentially prove one or more new taxa in the purple-flowered *Vincetoxicum* species complex. We cite representative specimens of these populations below.

##### Specimens examined.

**India.** Masrund, Chamba State, 21 June 1917, 5000 ft., *R.R. Stewart 2317* (RAW; MO); Bank of Ravi between Tiari & Siunn in dry places, Chamba State, 5000 ft., 04 July 1919, *R.N. Parker s.n.* (GH).

**Pakistan. Azad Jammu and Kashmir (AJK)**: Mountain slope near Deri Nala, 2500 ft., 06 May 2015, *S.A. Shah & B. Ali SAS-4* (RAW); Mandi, Kotli, 27 April 1954, *A. Rashid, E. Nasir, R.R. Stewart 27007* (RAW; BM); Kotli (Azad Kashmir), ±2700 ft., 03 June 1977, *Shahzad & Nisar 54346 & 54347* (ISL); Kotli (Azad Kashmir), 27 April 1954, *J. Muhammad 16636* (ISL); Doongi (Kotli), ±2500 ft., 01 June 1977, *Shahzad & Nisar 54341 & 54342* (ISL); Mausooh (Kotli, Azad Kashmir), 30 April 1977, *Shahzad & Nisar 50160*–*50162* (ISL); Nawal Nadi, Poonch, ±2700 ft., 18 June 1977, *Shehzad & Nisar 56415 & 56416* (ISL); Khohi Ratta (Kotli), 01 June 1977, *Shahzad & Nisar 54344 & 54345* (ISL); Muzaffarabad, ±3200 ft., 12 December 1975, *J. Muhammad 34527* (ISL); Zamanabad (Muzaffarabad), 25 April 1978, *S. Iqbal & W. Rehman 89644, 89646 & 89647* (ISL); Datta to Rara, (Muzaffarabad), 27 April 1978, *S. Iqbal & W. Rehman 89650 & 89651* (ISL); **Kashmir**: B-8 Pahlgam, ca. 8000 ft., s.d., *R.R. Stewart 5357* (K); Kashmir, Kullogam, s.d., *H. Falconer 2743* (K); Kashmir, Shapiyon, 7000 ft., s.d., *C.B. Clarke 28584 A, C* (K); Near Shapiyon, 6000 ft., s.d., *J.R. Drummond 13948* (K); Hab River banks, Lidar Valley, Kashmir, 06 June 1939, 7000 ft., *J.F. Ludlow 74* (BM); Mountains above Istahal River [Kashmir], 1915, 5000 ft., *Mrs. P. Decie s.n.* (BM); Tanmarg [Kashmir], s.d., *A.R. Naqshi 6343* (KASH); Sonamarg, Kashmir, 9200 ft., August 1928, *R.R. Stewart 13101* (MO); Kashmir, s.d., *H. Falconer* [?], s.n. (GOET [GOET020086]); **Khyber Pakhtunkhwa**: Darra, mountain slope, 3940 ft., 27 September 2016, *Faizan, A. Majid & S.A. Shah SAS-47* (RAW, US); Balakot (Kaghan valley), 6 July 1954, *Ch.S. Ali s.n.* (RAW); Balakot-Shogran Road, 28 June 1952, *I.I. Choudhri 13511* (RAW); Abbottabad, ca. 4500 ft., 01 June 1928, *R.R. Stewart 295* (KUH); Parhana [Abbotabad, KPK], 11 May 1976, *Shaukat & Nisar 18472* (ISL); Parhana, Hazara, 08 May 1976, *M.A Siddiqi, Shahzad, Ashraf, Manzoor, Maqsood & Dilawar 22587, 22588 & 22589* (ISL); Nari, 4 miles from Abbottabad, [KPK], 29 May 1976, *M.N. Chaudhri, M.A. Siddiqi, Shehzad, Ashraf, Maqsood, Lal & Akram 22597, 22598 &* –*22599* (ISL; GH[Col. No. 1122]); Bhonja [Mansehra], Hazara, 12 June 1976, *Shaukat & Nisar 22600 &22601* (ISL); Balakot, Hazara, 22 April 1978, *S. Iqbal & N. Ahmad 89641* (ISL); Shinkiari [Mansehra], 3500 ft., 30 May 1967, *E. Nasir, Siddiqi & Zaffar 4422* (KUH).

##### Representative specimens of the doubtful taxon Vincetoxicum sp. aff. arnottianum.

**Pakistan. Khyber Pakhtunkhwa**: Buner: Elum near Kalakhela, 860 m, 23 July 2015, *S.A. Shah & F. Rahman SAS-11* (RAW); Buner: On the way from Ghazi Kot to Mah Banr, 1700 m, 26 April 2015, *S.A. Shah & F. Rahman SAS-3* (RAW); Swat: Karakar, road side, 1300 m, 24 July 2015, *S.A. Shah SAS-12* (RAW); Mansehra: mountain slope near Bhonja village, 5740 ft., 28 September 2016, *S.A. Shah SAS-46* (RAW); Swat: Sherpalam, mountain slope under *Pinus* trees, 3310 ft. 10 July 2016, *S.A. Shah SAS-35* (RAW); Swat: Fizaghat road side near Darul Qaza, 1050 m, 10 May 2015, *S.A. Shah SAS-5* (RAW); Swat: Alam Ganj village, mountain slope, 1250 m, 20 July 2015, *S.A. Shah SAS-9* (RAW); Swat: Fatehpur, mountain slope, 2500 m, 28 September 2015, *S.A. Shah SAS-18* (RAW); Swat: Ghalegay, road side, 2790 ft, 07 May 2016, *S.A. Shah SAS-31* (RAW); Ayubia, pipeline track, 7545 ft., 04 September 2016, *R. Khan SAS-41* (RAW); Malakand: mountain slope near Butkhela bazaar, 2460 ft, 07 May 2016, *S.A. Shah SAS-29* (RAW); **Punjab**: Rawalpindi: Punjar, road side, 2250 ft., 15 May 2016, *S.A. Shah SAS-25* (RAW); Rawalpindi: Murree, Masyari, [6230 ft.], 01 August 2016, *Shakeel SAS-35* (RAW!).

#### 2. Vincetoxicum cabulicum

Taxon classificationPlantaeGentianalesApocynaceae

(Bornm.) Bornm. ex S.A. Shah

F15321EA-4920-59FA-98D0-109861D6339A

**Figure 7.**
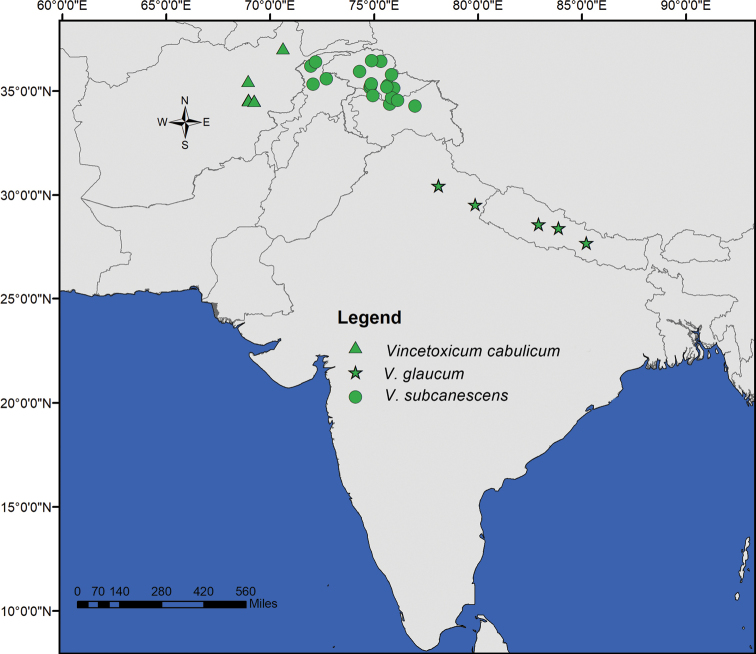
Geographic distribution of *Vincetoxicum cabulicum*, *V. glaucum* and *V. subcanescens*.

Figs 2K, L , 3B , 4C , 7

Cynanchum cabulicum Bornm., Bot. Jahrb. Syst. 66: 233. 1934.

##### Type.

Afghanistan. On Mt. Babour, 1800 m, 1 May 1929, *Manger s.n.* “flowers yellowish-green, inconspicuous” (***holotype*** B., destroyed). ***Neotype***, designated here: Afghanistan. Paghman, 7500 ft., dry slope; clumps; 3 ft. high; flr. greenish, often tinged madder; 26 June 1937, *W. Koelz 12076* (US! [US03264725]).

Undershrubs, ca. 45 cm tall. Stem striate, sub-canescent all around, internodes 2.5–7 cm long. Leaves subsessile, rarely petiolate; petioles 3–8 mm long, sub-canescent all around; lamina discolorous, narrow to broadly ovate, 3–7 × 2–4.5 cm, both surfaces including veins and margins sub-canescent; apex acuminate; margins smooth; base sub-cordate-round; veins visible on both surfaces, secondary veins up to 8 on each side of midvein. Inflorescences sessile or pedunculate; peduncles 1–2 cm long; bracts minute, ca. 1 mm long, margins ciliate; pedicels 3–6 mm long, sub-canescent; sepals tapering to acute apices, sub-canescent, ca. 1 mm long; corolla purple, corolla tube 1 mm long, lobes oblong-ovate, straight, 2–2.5 mm × 1–1.5 mm, pubescent within; corona lobes obovate, ca. 0.7–1 × 0.5 mm, divergent, as long as gynostegium. Follicles fusiform, ca. 5.3 × 1.3 cm. Seeds not seen.

##### Distribution and habitat.

Endemic to northern Afghanistan. The data on herbarium labels suggest that *V. cabulicum* grows in clumps on dry slopes of mountains. The elevation range is 1500 to 2800 m.

##### Phenology.

Flowering from May to June and fruiting from June to October.

##### Provisional conservation status.

Vulnerable (Table 1). *Vincetoxicum cabulicum* is collected from a few locations in Afghanistan and appears to be clumped in distribution. There are no reports on grazing. However, a possible threat appears to be habitat destruction through anthropogenic activities.

##### Notes.

This species was originally described as *Cynanchum cabulicum* by Bornmüller (1934). In the description, Bornmüller refers to “Syn. *Vincetoxicum cabulicum* Bornm. herb.”, thus creating an invalid name. Here, the name is validated as *Vincetoxicum cabulicum* (Bornm.) Bornm. ex S.A. Shah. Rechinger (1970) lumped this species with *V. glaucum* and, in turn, Ali and Khatoon (1982) lumped the latter with *V. canescens*. *Vincetoxicum glaucum* is hereby recognized as a separate species (see more comments on this complex below *V. subcanescens*).

##### Vernacular name.

Rang Koh-e-Sabz (Pashto).

##### Specimens examined.

**Afghanistan. Kabul**: Paghman, near Kabul, 7000 ft., 193[0s], *F. Howland specimen B.* (US [US03264726); Westhang des Koh-i-Sher Darwasa bei Kabul, 2100 m, 10 July 1969, *D. Podlech 15814* (RAW); Chandau, 8000 ft., 07 June 1937, *W. Koelz 11764* (US); In Valle Paghman, Kabul, ca. 34°36'N, 68°56'E, 2300–2800 m, s.d., K.H. Rechinger *17141* (US, MO); Kabul, in declivibus borealibus montis Scher Darwasa, ca. 34°30'N, 69°10'E, 1800–1900 m, s.d., K.H. Rechinger *16986* (US); Paghman, 8000 ft., 22 June [19]35, *W.R. Henry 273* (K); **Badakhshan**: Faisabad district, 5000 ft., 22 May 1964, *P. Furse 6247* (K).

#### 3. Vincetoxicum cardiostephanum

Taxon classificationPlantaeGentianalesApocynaceae

(Rech. f.) Rech. f., Fl. Iranica 73: 14. 1970

61230A92-9906-5E07-A18F-B8C593517ED1

**Figure 8.**
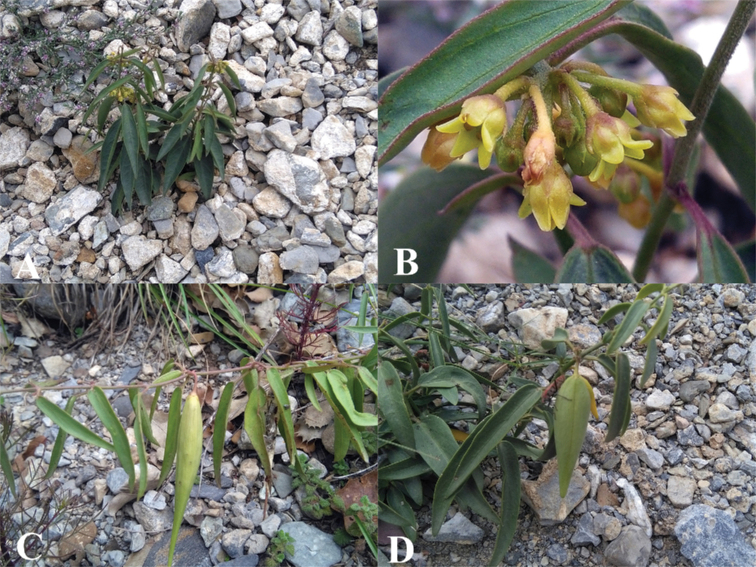
Field photographs of *Vincetoxicum cardiostephanum***A** habit **B** inflorescence **C, D** follicle. Photos by S.A. Shah.

**Figure 9.**
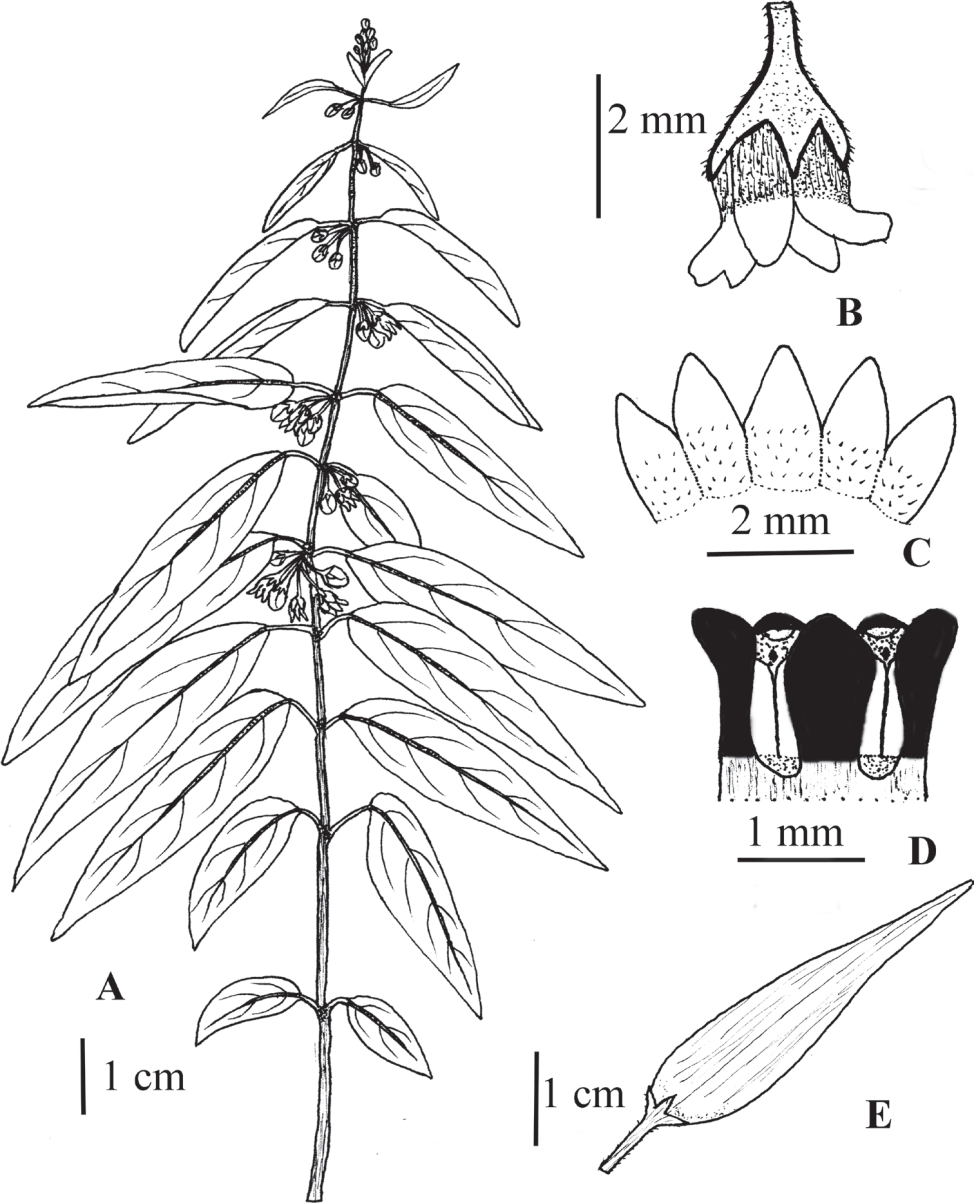
*Vincetoxicum cardiostephanum* illustrated by S.A. Shah & S.U. Nisa from *S.A. Shah, W. Hussain, Murtaza & M. Ullah SAS-40* (RAW!) **A** flowering plant **B** flower in natural orientation **C** corolla lobes **D** corona (black) **E** follicle.

Figs 2M, N , 3C , 4D, E , 6 , 8 , 9

Cynanchum cardiostephanum Rech. f., Österr. Akad. Wiss., Math.-Naturwiss. Kl. Anz. 105: 241. 1969

##### Type.

Afghanistan. Jaji, in declivibus jugi Narai Kotal versus Chakmani, in apertis quercetorum (*Qu. baloot*), substr. serpentin., 2100 m, 5 June 1967, K.H. *Rechinger 35614* (***Holotype***: WU online [WU 1969-0013837], ***Isotypes***: B online [B10 0365 118], US online [00112305]).

Small herbs to undershrubs, up to 40 cm tall. Stems striate, mostly pubescent along one or two lines, sometimes glabrous, internodes 1–4 cm long. Leaves dense, pendent; petioles 2–10 mm long, pubescent all around, sometimes only adaxial channel pubescent; lamina discolorous, narrowly lanceolate-ovate, 3–7 × 0.6–2 cm, both surfaces glabrous; apex acute to sometimes narrowly acute; base mostly obtuse; veins visible on both sides, sometimes inconspicuous, secondary veins up to 8 (–10) on each side of midvein, adaxial veins sparsely to densely pubescent, abaxial veins glabrous to glabrescent; margins sparsely pubescent. Inflorescences both sessile and shortly pedunculate; peduncles up to 6 mm long; bracts narrow, up to 4 mm long, margins ciliate; pedicels 2–5 mm long, pubescent along one line. Flowers 2.5–3 × 1.5–2 mm, pendent; sepals tapering to acute apices, up to 1 mm long, laterally sparsely ciliate, calycine colleters paired (10/flower); corolla yellowish-green, campanulate, corolla tube prominent, 1 to 1.5 mm long, lobes oblong-ovate with obtuse apices, 1–1.5 × 1 mm; corona clavate, ca. 0.7 × 0.6 mm, slightly exceeding the gynostegium in length, divergent. Follicles ovate-lanceolate to narrowly fusiform, up to 7 × 1 mm, apex acuminate, glabrous. Seeds dark brown, ca. 7 × 3 mm, wings up to 1 mm broad; coma up to 2 mm long.

##### Distribution and habitat.

Two collections of this species are from Shalozan, Kurram valley, Pakistan. This place is located on the eastern border of Afghanistan. The type specimen is collected from Khost, Afghanistan, which is located nearby Kurram Valley. The habitat of the plant is open mountain slopes consisting of small stones and gravel. Elevation ranges from 2100 to 2200 m.

##### Phenology.

*Vincetoxicum cardiostephanum* flowers from July to August and fruits from August to October.

##### Provisional conservation status.

Critically endangered (Table 1). *Vincetoxicum cardiostephanum* is extremely rare and comprises very small populations of fewer than 50 individuals. It is known only from three localities in Pakistan and Afghanistan. It was declared critically endangered by Hussain et al. (2019). Erosion is the biggest threat for this species. We have successfully potted a plant in NARC, Islamabad. This indicates that the plant could be conserved in ex-situ conditions.

##### Notes.

In the herbarium (RAW), only one gathering of this species from Kurram Valley, Pakistan was available, collected by Harsukh in 1894 and filed under *Cynanchum vincetoxicum* (syn. *V. hirundinaria*). We re-discovered the species from the same area after 122 years. The population was composed of a mere 15 individuals. The type gathering of this species was collected from the adjacent area in Afghanistan.

##### Specimens examined.

**Pakistan. Khyber Pakhtunkhwa**: Kurram Valley, 1894, *Harsukh 15402* (RAW, K); Parachinar: near Khaiwas in Shalozan valley, 2200 m, 07 August 2016, *S.A. Shah*, *W. Hussain, M. Hussain, M. Ullah SAS-40* (RAW, US).

#### 4. Vincetoxicum glaucum

Taxon classificationPlantaeGentianalesApocynaceae

(Wall. ex Wight) Rech. f., Fl. Iranica 73: 13. 1970

8A68959E-3719-5A94-A671-391CF3B51519

Figs 3D , 4F , 7

Cynanchum glaucum Wall. ex Wight, Contr. Bot. India: 58. 1834. Vincetoxicum hirundinaria subsp. glaucum (Wall. ex Wight) H. Hara, Enum. Fl. Pl. Nepal 3: 89. 1982.

##### Type.

Nepal. Chandaghir, 5 May 1821, *N. Wallich 133* [cat. #. 8229A] (***lectotype***: designated by Hara (1982), pointing generally to the set of syntypes: Asclep 133 and cat. # 8229A, specimen not chosen from syntypes); ***lectotype*** (designated here: K online [K000894587]; ***isolectotypes*** K online [K001129297, K000894586]; E online [E00179664, E00179665]).

Undershrubs, up to 40 cm tall. Stem striate, pubescent all around, internodes 1–6.8 cm long. Leaves petiolate; petioles 3–11 mm long, pubescent all around; lamina different shaped: narrowly ovate, oblong-ovate, elliptic ovate, lanceolate-ovate, 4–9.2 × 1.3–3.8 cm; margins smooth; apex acute to obtuse or sometimes mucronulate; base round or cuneate; veins visible on both surfaces, secondary veins 8–12 on each side of midvein; adaxial surface sub-glabrous, adaxial veins densely pubescent; abaxial surface glabrous to sub-glabrous, abaxial veins especially midrib pubescent; margins pubescent. Inflorescences sessile; bracts linear, ciliate; sepals tapering to acute apices, 1.5 mm long with ciliate margins; pedicels 1–3 mm long, pubescent; calycine colleters 5, exceeding the corolla tube in length; corolla green, not twisted, corolla tube 1 mm long, lobes tapering to pointed apex, 2 × 1 mm, bearded within; corona lobes longer than broad, 1 × 0.8 mm, exceeding the length of the gynostegium, base narrow, apex broad, toothed, divergent; staminal appendages obtuse; pollinaria deeply embedded in the gynostegium. Follicles and seeds not seen.

##### Distribution and habitat.

Endemic to eastern Himalayas including India and Nepal and occurring at an elevation of over 2000 m. Herbarium label on *W. Dudgeon & L.A. Kenoyer 56* (MO) indicates its habitat to be open grassy places.

##### Phenology.

Flowering from May to June and fruiting from July to October.

##### Provisional conservation status.

Data Deficient (Table 1). *Vincetoxicum glaucum* is collected from a few localities in the eastern Himalayas (India and Nepal). We did not collect or observe its populations in natural habitats. Herbarium labels do not provide significant information about its populations. Therefore, it is declared as data deficient.

##### Notes.

*Vincetoxicum glaucum* was first described as *Cynanchum glaucum* Wight but soon regarded as *V. canescens* by Decaisne (1844), a treatment which Boissier (1879) also adopted. Hooker (1883) reinstated the original name *C. glaucum*. Rechinger (1970) recognized *V. glaucum* in a broader sense and lumped with it another Afghani species, *V. cabulicum* Bornm. Hara et al. (1982) regarded Rechinger’s *V. glaucum* as a synonym of V. hirundinaria subsp. glaucum (Wall. ex Wight) H. Hara. While writing the genus *Vincetoxicum* for the flora of Pakistan, Ali and Khatoon (1982) lumped Rechinger’s *V. glaucum* with *V. canescens*. In the present treatment, we recognize three different species in this long misunderstood species complex, namely *V. cabulicum* (endemic to northern Afghanistan), *V. glaucum* (endemic to India and Nepal) and *V. subcanescens* sp.nov. (endemic to Pakistan, Kashmir and Tibet), whereas *V. canescens*, a species long regarded as a member of this complex, is endemic to the eastern Mediterranean region and does not occur in southern Asia (also see notes below *V. subcanescens*).

Wight (1834) cited Wallich’s collection Asclep. # 133 (Cat. # 8229A) as *C. glaucum* (corresponding to the typical variety *latifolium*, denoted by “α” in the protologue), Asclep. # 132 as variety *oblongifolium* (denoted by “β” in the protologue), and Asclep. # 134 and Cat. # 1554 as variety *lanceolatum* (denoted by “γ” in the protologue). He did not designate a holotype for *C. glaucum*, hence the specimens cited in the protologue and their duplicates were the syntypes for the respective varieties. Later on, Hara (1982) lectotypified *C. glaucum* by designating Wallich’s Asclep. # 133 (Cat. # 8229A). This collection has at least five duplicates (syntypes) housed by K [K000894587, K001129297, K000894586] and E [E00179664, E00179665]). Possibly, Hara considered Asclep. # 133 a single specimen and did not choose a specimen as lectotype from the duplicates (syntypes). In spite of Hara’s attempt, the lectotype is still unknown among the syntypes. Therefore, it is necessary to choose a specimen from the five duplicates (syntypes) of Wallich’s Asclep. # 133 (Cat. # 8229A). Among them, K000894587 is chosen here as the lectotype of *C. glaucum*. The remaining syntypes become isolectotypes. We could not get specimen loans and examine the collections Asclep. # 132 & 134 and Cat. # 1554 that belong to the remaining two varieties of *C. glaucum*. Therefore, we recommend a revision for these varieties. It is also notable that there is no mention of these varieties in the post-protologue literature.

##### Specimens examined.

**India.** Uttarakhand: Landour, open grassy places, 7000 ft, 24 May 1920, *W. Dudgeon & L.A. Kenoyer 56* (MO [MO-2321710]); Nepal, s.d., *N. Wallich* cat. # 8229A (K [K001129296]); Kumaon/Tranquebar, India, s.d., *N. Wallich* cat. # 8229B (K [K001129299]).

#### 5. Vincetoxicum kenouriense

Taxon classificationPlantaeGentianalesApocynaceae

(Wight) Wight, Icon. Pl. Ind. Orient., pl. 1614. 1850

F0417E9C-EF27-58D7-AA12-1685442084C3

**Figure 10.**
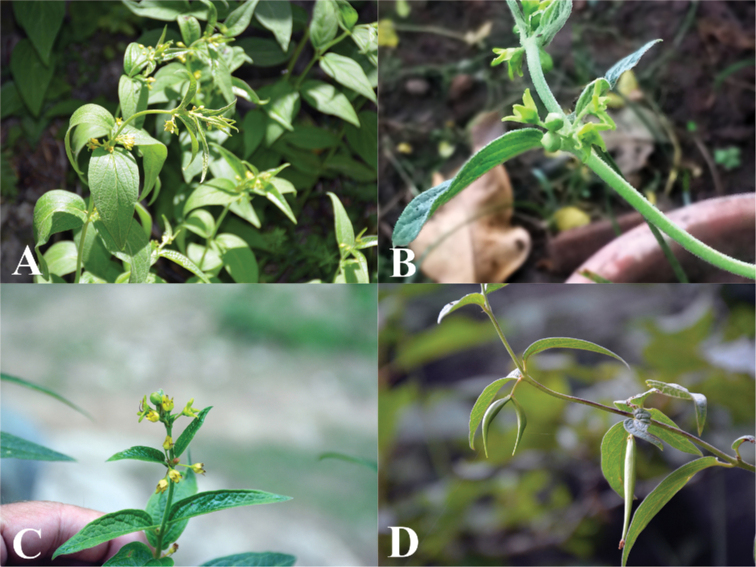
Field photographs of *Vincetoxicum kenouriense***A** habit **B, C** flowers **D** follicles. Photos by Zahid Ullah and S.A. Shah.

**Figure 11.**
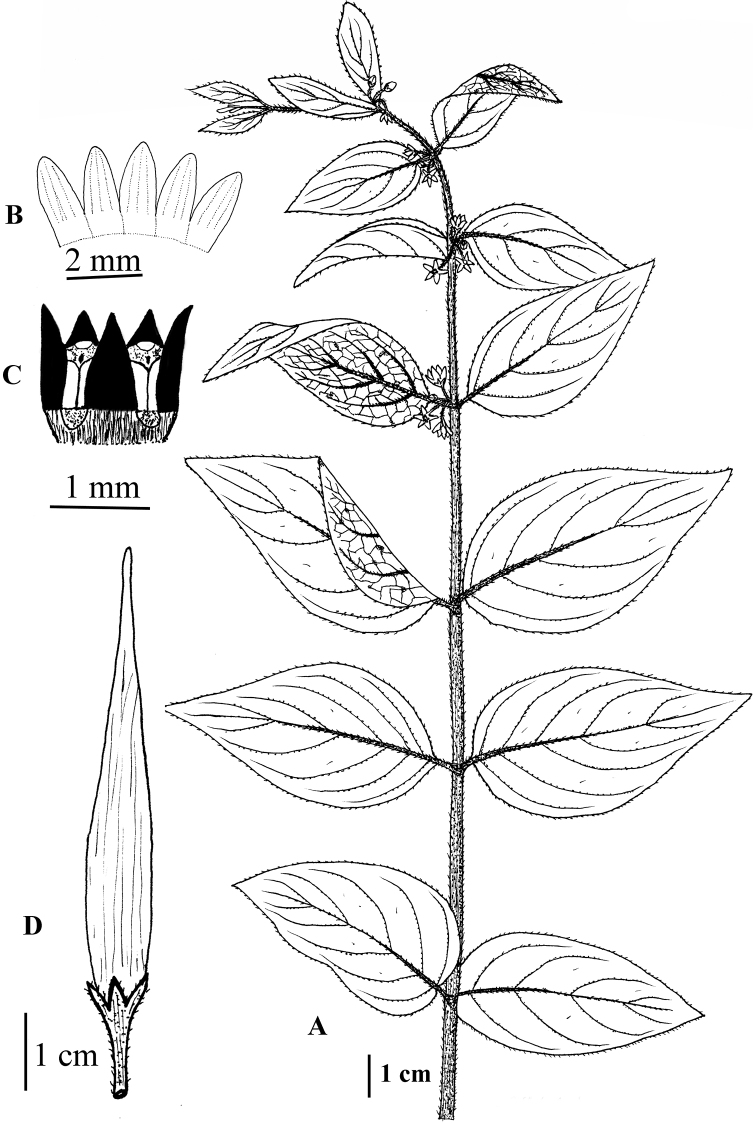
*Vincetoxicum kenouriense* illustrated by S.A. Shah and S.U. Nisa from *S.A. Shah & L. Ahmad SAS-37* (RAW!) **A** flowering plant **B** corolla lobes **C** corona (black) **D** follicle.

**Figure 12.**
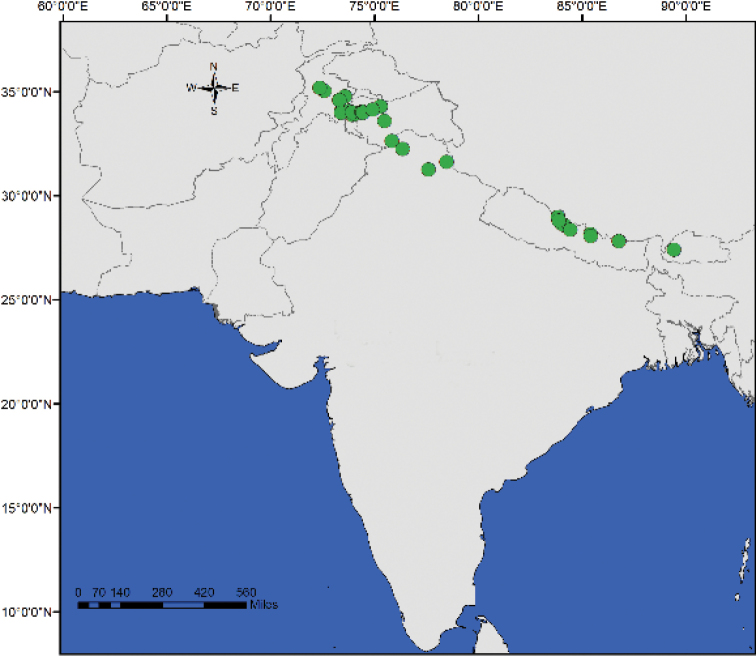
Geographic distribution of *Vincetoxicum kenouriense*.

Figs 2O, P , 3E , 4G, H , 10 , 11 , 12

Cynanchum kenouriense Wight, Contr. Bot. India 58. 1834.

##### Type.

India. Kenour, *Royle 18* (***Holotype*** K! [K000872737]).

Undershrubs, up to 1 m tall. Stem pubescent all around or along 2 dense lines, internodes 2–13 cm long. Leaves opposite; petioles 1–10 (–14) mm long, equally pubescent all around or sometimes denser along adaxial channel, lamina strongly discolorous, narrow to broadly ovate, 4–10 × 1.8–6.5 cm; apex narrowly acute to very shortly acuminate; base round to sub-cordate to sub-truncate; venation including tertiary and quaternary veins prominent, more prominent and raised on abaxial surface, secondary veins up to 14 on each side of midvein, trichomes absent on both surfaces, sometimes sparsely present on tertiary and quaternary veins, midrib and secondary veins densely pubescent on both surfaces, margins pubescent. Inflorescences mostly sessile, rarely both sessile and short-pedunculate inflorescences present on the same plant, peduncles up to 1.5 cm; bracts linear, up to 2 mm long, pubescent; pedicels 2–8 (–10) mm long, pubescent all around, sometimes pubescent along one or two longitudinal lines. Flowers yellowish-green to green, 4–5 × 2–2.5 mm; sepals tapering to acute or narrowly acute apices, up to 2 mm long, margins sparsely ciliate, calycine colleters 5 or 10 per flower; corolla tube ca. 1 mm long, lobes oblong with obtuse apices, 3–3.5 × 1–2 mm, glabrous within or sparse caducous trichomes present; corona lobes long-triangular with acute apex, slightly divergent, ca. 1 × 0.8 mm, exceeding slightly the length of the gynostegium. Follicles fusiform, up to 7 × 1 cm, apex long-acuminate, surface glabrous, slightly striate. Seeds brown, 6–7 × 3–3.5 mm, wings less than 1 mm broad; coma 2–3 mm long.

##### Distribution and habitat.

Endemic to a long geographic range in the Hindukush Himalayas from Bhutan in the east to Pakistan in the west. The western limit of this species is district Swat (Khyber Pakhtunkhwa province), Pakistan. It occurs on higher elevations between 1500 and 3000 m. The habitats of the species are commonly the Himalayan moist temperate forests (evergreen forests of conifers between 1500 to 3000 m elevations), rarely subalpine or open alpine lands (above 3000 m). We collected it from open stony alpine slopes and stream side slopes in a V-shaped mountain valley. The associated vegetation was either alpine herbs or conifers, and herbaceous to shrubby flora.

##### Phenology.

Flowering from May to August and fruiting from August to October.

##### Provisional conservation status.

Least concern (Table 1). Although having highly clumped, distant populations, *Vincetoxicum kenouriense* spans a wide range in the Hindukush Himalayas. It is confined to higher altitudes and faces almost no natural threats. Anthropogenic activities, however, are deemed as a potential threat. For instance, two of its populations in Pakistan (in district Bagh, AJK, and district Swat, Pakistan) were found in recreational areas that are expected to undergo changes in the near future which might result in destruction of its habitats. It was also collected previously from Changla Gali (district Abbotabad, Pakistan). In spite of several visits in the past decade, we could not find it anymore in that area. These observations suggest that recreational activities might prove a potential threat to its existence, at least in Pakistan.

##### Notes.

*Vincetoxicum kenouriense* was described by Wight (1834) from Kenour (India). Hooker (1883) synonymized it with *Cynanchum vincetoxicum* (syn. *V. hirundinaria*). Hooker’s treatment has been followed in major floras (Stewart et al. 1972; Ali and Khatoon 1982) resulting in a broader circumscription of *V. hirundinaria*. In the present treatment, *V. kenouriense* is reinstated and presented as a new record for the Flora of Pakistan, Nepal and Bhutan. It replaces *V. hirundinaria* in southern Asia.

##### Specimens examined.

**Bhutan. Thimpu**: Paro (West Bhutan), 7800 ft., 27 June 1933, *F. Ludlow & G. Sherriff 158* (BM [BM001119300]).

**India. Uttarakhand**: Garhwal, Mussoorie, Kidar Kantha, 13000 ft., 09 April 1952, *J.R. Drummond 22753* (K); Hab. Sikkim, 7000–10,000 ft., s.d., *J.D. H[ooker] s.n.* (GH, [GH01147527]); Daya-Balsan state, Simla hills, 7500 feet, 12 June 1937, *G.E. Parkinson 7386* (RAW); Dharmsala [India], 17 June 1929, [*R.R. Stewart] s.n.* (RAW [acc. # 1004]); s.loc., s.d., *H. Falconer s.n.* (GH [GH01147530]); Himalaya Bor. Oce., 4000–5000 ft., s.d., *W. Griffith* 3760 (GH [GH01147532]).

**Kashmir**: B-8 above Gulmarg, 9000–10,000 ft., 31 July [18]92, *J.F. Duthie s.n.* (K); Basaoli, 8000 ft., s.d., *C.B. Clarke 31523A* (K); Tanmarg near Gulmarg, Kashmir, 02 September 1929, *R.R. Stewart 13100* (RAW); Sonamarg, 9000 ft., s.d., *R.R. Stewart 7306* (K); Below Sonamarg, Sindh Valley, 8000–9000 ft., 02 September 1940, *R.R. Stewart 21328* (RAW, US); Gulmarg to Khaipur, 21 July 1890, *J.F. Duthie s.n.* (RAW [acc. # 1005]); Duksum-Kokernagh [Kashmir], 3000 m, s.d., *G.A. Shapu 201* (KASH); Slopes above Harwan [Kashmir], 2300 m, s.d., *G. Singh 833* (KASH).

**Nepal. Gandaki Zone**: Manang Distr. Pisang (3090 m), Humre (340 m), Manang (3360 m), 16 August 1994, *M. Mikage, N. Fujii, T. Kajita, N. Kondo, S. Noshiro & K.Yoda, 9485444* (GH); **Mustang District**: Gnyu Pass (4100 m), Chhengar (3700 m), Muktinath (3650 m), 14 July 2000, *Y. Lokawa, M.N. Subedi, Y. Takashi & K. Kano 20020202* (GH); On descendant and l’lmja-Khola, 3100 m, 09 April 1952, *A. Zimmermann 714* (K); Baghmati zone, before Syarpagaon, north side of Lantang River, 2600 m, 19 September 1966, *D.H. Nicolson 2445* (US); **Bagmati zone**: south of Gossainkunde, Blumche, 2500 m, 12 May 1967, *D.H. Nicolson 3339* (US).

**Pakistan. Khyber Pakhtunkhwa**: B-7 Hazara, Kaghan valley, 9800 ft., 09 July 1897, *Inayat 19940* (K); Nathia, July 1907, *H. Deane s.n.* (K); Mundi, UNA forest, Siran Valley, District Mansehra, 13 June 1994, *Q. Marwat 425* (RAW); C-7 Murree Hills Changlagali, 8000 ft., *M. Nath 336* (RAW); **Azad Jammu and Kashmir**: Bagh, 19 June 1956, *collector unknown 1507* (KUH); Bagh, Sudhan Gali to Ganga Choti, 2500–2800 m, 01 August 2016, *S.A. Shah & L. Ahmad SAS-37* (RAW, US); Bagh, Poonch, 19 June 1956, *M.A. Kazmi 1507* (RAW); C-8 Azad Kashmir, Poonch, near Trappar, below Kali, 17 September 1952, *A.R. Khan s.n.* (RAW [acc. # 998]); Raikot to Aliabad (Azad Kashmir), ±7000 [ft], 28 June 1952, *R.R Stewart & E. Nasir 23868* (KUH); Azad Kashmir, 8000 ft., 9 August 1969, *Shariq 8102* (PFI); Pir Kanthi, Uri Range, 9000 ft., 17 October 1955, *J. Mohammad s.n.* (RAW).

#### 6. Vincetoxicum lenifolium

Taxon classificationPlantaeGentianalesApocynaceae

S.A. Shah sp. nov.

09F3BB25-ACED-50AC-9089-8EC921730350

urn:lsid:ipni.org:names:77217788-1

**Figure 13.**
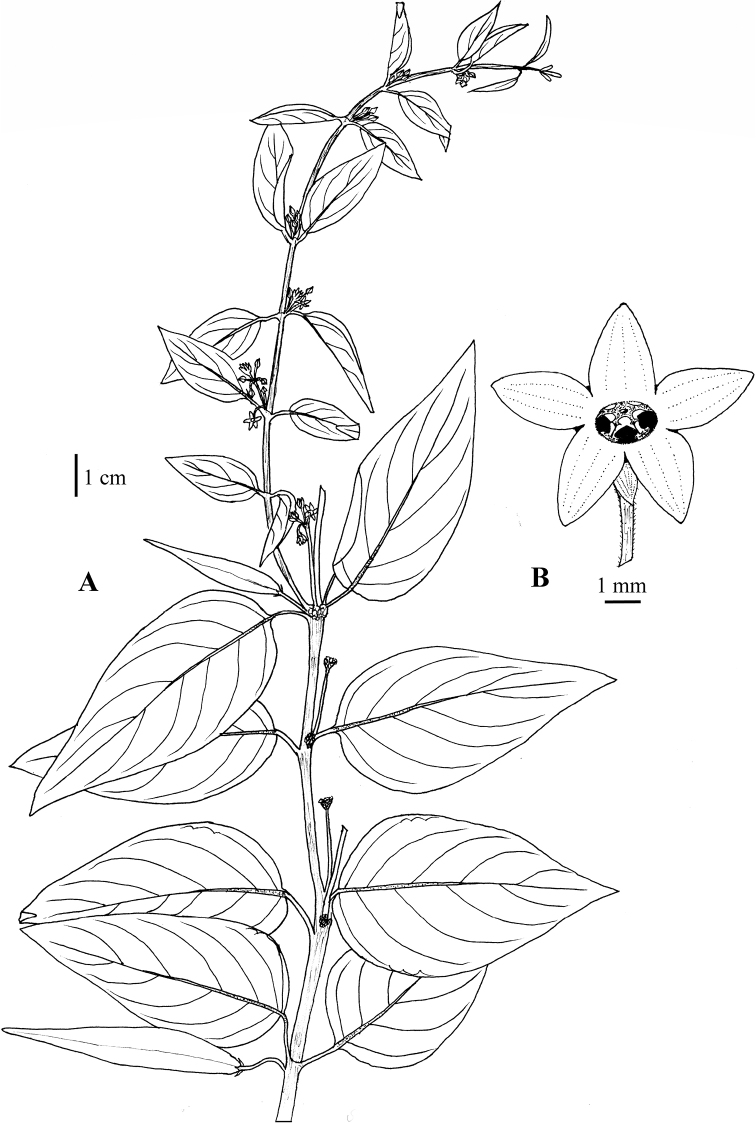
*Vincetoxicum lenifolium* illustrated by S.A. Shah & S.U. Nisa from *N. Ali 1035* (RAW) **A** plant with fruits and flowers **B** flower, top view.

Figs 2Q, R, U, V , 3F , 4I, J, M , 6 , 13

##### Diagnosis.

Differing from *V. stocksii* by having broader (2.5–4.7 × 1–4.5 cm), ovate leaves with lamina glabrous to inconspicuously puberulent, green flowers, internally glabrous corolla lobes, and small, ca. 0.7 mm long, usually rhomboid corona lobes. In *V. stocksii*, leaves are narrowly ovate to narrowly or broadly lanceolate, rarely elliptic-ovate (3–6 × 1–2 cm) with lamina sparsely pubescent on both sides, flowers bicolored, corolla lobes pilose within, and corona lobes subulate and longer than the gynostegium (ca. 1 mm long).

##### Type.

Pakistan. Razmak [North Waziristan], 193[0s], *N. Ali 1035* (***Holotype***: RAW).

Undershrubs, ca. 50 cm tall. Stem striate, pubescent along one or two lines, rarely all around, internodes 2–7 cm long. Leaves opposite; petioles 4–15 mm long, pubescent all around or only along the adaxial channel; lamina discolorous, sometimes seemingly unifacial, lanceolate-ovate to ovate, 2.5–4.7 × 1–4.5 cm, spreading, sometimes pendent; apex acute to narrowly acute to shortly acuminate; base round or sub-cordate; veins prominent on both surfaces, secondary veins 8–10 (–14) on each side of midvein, both adaxial and abaxial surfaces mostly glabrous to inconspicuously puberulent, adaxial veins densely pubescent, abaxial veins and lamina margins glabrescent. Inflorescences long-pedunculate in the lower nodes to sessile in the upper nodes; peduncles up to 2.5 cm long, puberulent all around or along 1–2 lines; bracts linear, 2 mm long. Flowers green, ca. 3 × 2 mm; pedicels 2–5 mm long; sepals tapering to acute or narrowly acute apices, ca. 1.5 mm long, margins ciliate, abaxial surface sometimes pubescent, calycine colleters 10 per flower; corolla tube ca. 1 mm long, lobes oblong-ovate with obtuse or emarginate apex, 1.5–2.5 × 1–1.3 mm, glabrous within; corona lobes rhomboid, sometimes variable shapes found: conical-rhomboid, deltoid-rhomboid, rarely elongated, almost equal in length and width, 0.4–0.7 × 0.4–0.8 mm, reaching the base of the staminal appendages, erect to slightly divergent; staminal appendages obtuse. Follicles fusiform, 5–8 × 0.4–0.7 cm, apex acuminate, surface glabrous to glabrescent, striate. Seeds light brown, ca. 4 × 2.2 mm, wings less than 1 mm broad; coma up to 2 cm long.

##### Distribution and habitat.

Endemic to Khyber Pakhtunkhwa province of Pakistan. So far, it has been recorded from North & South Waziristan and Kohat districts. The elevation ranges from 1500 to 2000 m. Its habitat is open rocky slopes consisting of small stones and gravel and stream beds.

##### Etymology.

The name is based on smooth, mostly glabrous, leaves of the species.

##### Phenology.

Flowering from April to June and fruiting from July to August.

##### Provisional conservation status.

Endangered (Table 1). *Vincetoxicum lenifolium* is endemic to a small range comprising three districts of Khyber Pakhtunkhwa province, Pakistan. In 2016, we collected fresh specimens and observed its population structure in Razmak (North Waziristan). The population was clumped with less than 20 individuals. Anthropogenic activities like land degradation, settlements, roads etc. are deemed major threats to its existence.

##### Notes.

From the general appearance, *V. lenifolium* appears as a closely related member of the purple-flowered *Vincetoxicum* including *V. arnottianum*, *V. luridum*, *V. sakesarense* and *V. stocksii*. The paratypic specimens of this species were hitherto misidentified as *V. arnottianum* or *V. hirundinaria*. However, the new species is easily distinguished by ovate leaves, green flowers and glabrous corolla from *V. arnottianum* and by inconspicuous veins, denser inflorescences and small rhomboid corona from *V. hirundinaria*. RAW houses a variable specimen, N. Ali 1029, which is silvery in appearance, with sessile inflorescences and corona lobes somewhat deltoid (Fig. 3H). These characters and the associated geographic information do not support the recognition of the specimen as a separate taxon. The specimen is cited as *V. lenifolium* in this treatment. Long-pedunculate inflorescences are most commonly found in *V. lenifolium* and rarely in *V. stocksii*. Short-pedunculate along with sessile inflorescences are found in *V. arnottianum*, *V. cabulicum*, *V. cardiostephanum*, rarely in *V. kenouriense* and *V. sakesarense*, *V. luridum*, *V. stocksii*, and *V. subcanescens*. Sessile inflorescences are found in *V. glaucum* and *V. stewartianum*.

##### Specimens examined.

**Pakistan. Khyber Pakhtunkhwa**: Kaniguram [South Waziristan], 13 May 1895, *J.F. Duthie 15766* (RAW); Paryat (N. Waziristan), ±5000 ft., 01 June 1979, *M. Zubair & S. Khan 114964 & 114967* (ISL); Enger (North Waziristan), 21 June 1977, *H. Ullah & Ayaz 56417 & 56418* (ISL); North Waziristan: Razmak, sandy slope on roadside near Razmak bazaar, 7545 ft., 08 September 2016, *S.A. Shah SAS-44* (RAW US); Razmak, 29 May 1979, *M. Zubair & Dilawar 112288* (ISL); Razmak, ±6500 ft., 29 May 1979, *M. Zubair & S. Khan 112298* (ISL); Razmak [North Waziristan], 193[0s], *N. Ali 1029* (RAW!); Togh Sarai, Kohat [KPK], 31 March 1979, *M. Zubair & S. Khan 113954* (ISL).

#### 7. Vincetoxicum luridum

Taxon classificationPlantaeGentianalesApocynaceae

Stocks ex S.A. Shah

DFEC6FF3-779A-55AB-A8D7-68789DD8D85A

Figs 2S, T , 3G , 4K, L , 6

##### Type.

Pakistan. Balochistan: “Balochistan”, 3500–5500 feet, [1849], [*Stocks] 721* (***Holotype*** K! [K001235295], isotypes: K! [K001235294], [K001235296]).

See more details in Shah et al. (2018).

#### 8. Vincetoxicum sakesarense

Taxon classificationPlantaeGentianalesApocynaceae

Ali & Khatoon, Pak. J. Bot. 14(1): 67. 1982

AD7FA519-AF41-5E2E-9EDB-AE658604AB98

Figs 2W, X , 3I , 4N, O , 6

##### Type.

Pakistan. Punjab: C-7 Sargodha Dist.: Sakesar hills, in protected area, 15.8. [19]72, *M. Qaiser & A. Ghafoor* 4524 (***Holotype***: KUH!).

Undershrubs, ca. 55 cm tall. Stem striate, pubescent along single or double lines, sometimes pubescent all around, internodes 1–7 cm long. Leaves opposite; petioles up to 8 mm long, pubescent along adaxial channel, sometimes sparsely pubescent abaxially; lamina narrowly ovate to lanceolate, 3–11 × 1–3 cm; apex acute to narrowly acute; base round to sub-cuneate, veins visible on both sides, secondary veins 6–7 (–12) on each side of midvein, both adaxial and abaxial surfaces and abaxial veins glabrous, adaxial veins pubescent, margins sparsely pubescent. Inflorescences sessile, rarely small peduncles up to 2 mm present; bracts with a few basal trichomes or completely glabrous. Flowers dark purple except sepals, 4 × 2.5 mm; pedicels 2–5 mm long, puberulent; sepals up to 2 mm long, tapering into narrowly acute to acuminate apices, margins ciliate, abaxial surface sometimes pubescent, calycine colleters 10/flower; corolla tube up to 1 mm long, lobes 2.5 × 1 mm, twisted clockwise, gradually tapering to the apex, inner surface of corolla lobes bearded except one lateral margin; corona lobes deltoid, longer than wide, 0.6 × 0.5 mm, almost reaching the bases of the staminal appendages, divergent. Follicles fusiform, 5.5–6.5 × 0.7 cm, apices acuminate, surface inconspicuously striate, glabrous. Seeds not seen.

##### Distribution and habitat.

Endemic to a protected area in Soon Sakesar Valley, a small mountainous valley in northern Punjab, Pakistan. The maximum elevation is 1500 m.

##### Phenology.

Flowering from April to September and fruiting from May to October.

##### Provisional conservation status

**(Table 1).** Critically endangered. *Vincetoxicum sakesarense* is known from two localities in Soon Sakesar valley, Salt Range, Punjab province, Pakistan. The area is protected for security purposes. We and other botanists have collected the species from the type locality twice in the current decade. The species is critically endangered due to the fact that its EOO is very small (Table 1), and that it is confined to a comparatively small mountain range. Expanding human population coupled with anthropogenic activities are a serious threat. We have grown living plants in NARC, Islamabad. This indicates that this species could potentially be conserved outside its native range.

##### Notes.

*Vincetoxicum sakesarense* is a member of the purple-flowered group of *Vincetoxicum* and most closely related to *V. arnottianum*. The two species can be readily distinguished by the shape of the corona lobes which is deltoid in *V. sakesarense* and deltoid-rhomboid in *V. arnottianum*. Although very similar, *V. arnottianum* occurs in western Himalaya while *V. sakesarense* occurs in the Salt Range (non-Himalayan Mountains).

##### Specimens examined.

**Pakistan. Punjab**: Sakesar, 29 September 1951, *A. Rahman 287* (KUH); Sakesar, 27 April 1977, *M. Ajab & M. Ashraf 50159* (ISL); Sakesar, 05 May 1978, *M. Ajab & M. Ahmad 83837* (ISL); Sakesar, 27 April 1977, *M. Ajab & M. Ashraf 50158* (ISL); Ouchali, 28 April 1977, *M. Ajab & M. Ashraf 48440* (ISL); Sakesar, 01 August 1954, *I. Ahmad 27821* (RAW).

#### 9. Vincetoxicum stewartianum

Taxon classificationPlantaeGentianalesApocynaceae

S.A. Shah sp. nov.

97294066-A901-52FC-802E-8D87DEA92572

urn:lsid:ipni.org:names:77217789-1

**Figure 14.**
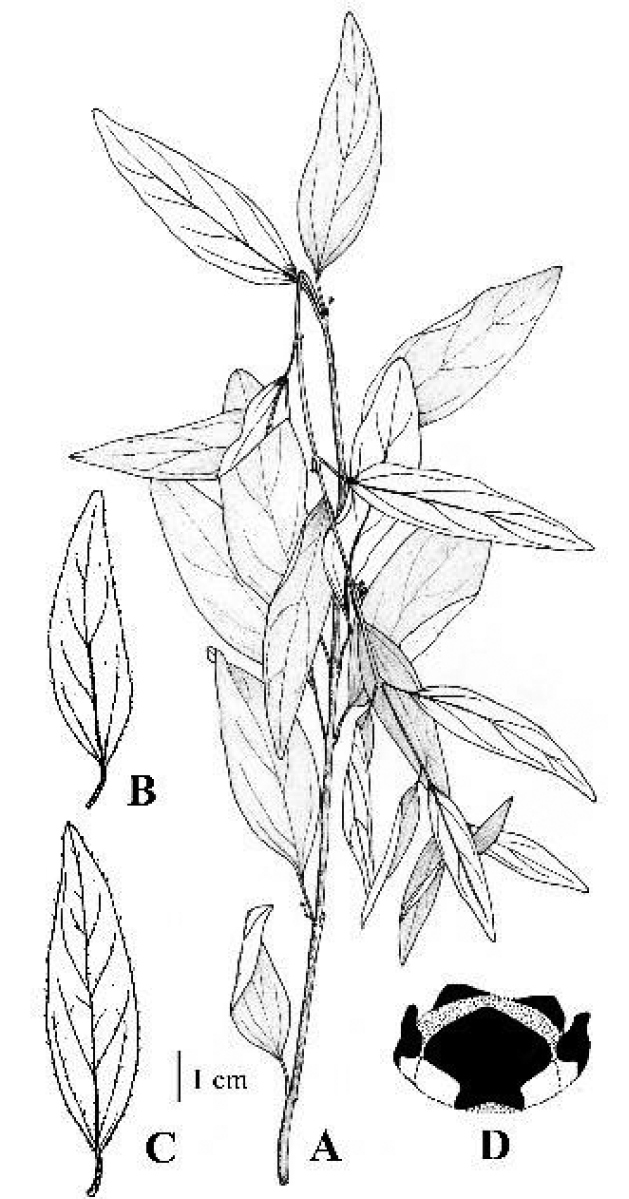
*Vincetoxicum stewartianum* illustrated by M. Saleem from *R.R. Stewart 16709* (RAW) **A** plant **B** adaxial surface of leaf **C** abaxial surface of leaf **D** corona.

Figs 2Y, Z , 3J , 4P , 6 , 14

##### Diagnosis.

Differing from *V. arnottianum* by having retrorse indumentum on the stems, ovate to elliptic-ovate leaves, light purple flowers, and large rhomboid (1 × 1 mm) corona lobes that exceed the gynostegium in length. In *V. arnottianum*, the indumentum is extrorse on the stems, leaves are mostly narrowly ovate or lanceolate-ovate, flowers dark purple, and corona lobes are deltoid-rhomboid and do not exceed the gynostegium in length.

##### Type.

India. Uttarakhand: Landour, Mussoorie, 6–7000 ft. 8 August 1938, *R.R. Stewart 16709* (***Holotype***: RAW!).

Undershrubs, ca. 50 cm tall. Stem striate, pubescent all around, trichomes retrorse, internodes 1.5–5 cm long. Leaves opposite, petiolate; petioles 2–15 mm long, sparsely pubescent all around; lamina discolorous, ovate- or elliptic-lanceolate to narrowly ovate, 5–7.5 × 2–3 cm; apex acute to obtuse sometimes mucronulate; base obtuse to sub-obtuse; veins visible on both sides, secondary veins 8–14 on each side of midrib, both adaxial and abaxial surfaces of lamina glabrous to sometimes sub-glabrous, veins on both surfaces and margins pubescent. Inflorescences sessile; pedicels 2–3 mm long, pubescent along a narrow longitudinal line; bracts narrow tapering, pubescent. Flowers light purple, ca. 5 × 2 mm; sepals tapering to acute apices, up to 2 mm long with ciliate margins, calycine colleters single; corolla tube ca. 1 mm long, lobes oblong, narrowly tapering, ca. 3 × 1–2 mm, bearded within; corona lobes rhomboid, ca. 1 × 1 mm, exceeding the length of the gynostegium, divergent. Follicles and seeds unknown.

##### Distribution and habitat.

Endemic to eastern Himalayan Mountains of India. The elevation range is 6000 to 7000 m. The limited herbarium materials do not provide more information.

##### Phenology.

*Vincetoxicum stewartianum* flowers and fruits from August to September.

##### Provisional conservation status.

Data deficient (Table 1). *Vincetoxicum stewartianum* is gathered from only the type locality. Besides label data on gatherings from the type locality, information is lacking about its present occurrence. A survey on its occurrence and ecology is recommended.

##### Etymology.

Named after the collector Prof. R.R. Stewart.

##### Notes.

The specimens of this species were hitherto misidentified as *V. glaucum* and *V. canescens*.

##### Specimens examined.

**India.** Himal. Bor. Occ.; Regio temp., s.d., *T. Thomson* s.n. (GH [GH01147533]; GOET [GOET020096]).

#### 10. Vincetoxicum stocksii

Taxon classificationPlantaeGentianalesApocynaceae

Ali & Khatoon., Pak. J. Bot. 14(1): 65. 1982

E048150D-2411-5071-A434-EDE9BB3CAC11

##### Type.

Pakistan. Balochistan: D-5 Near Bharat Khel on way to Zhob, erect shrub, l m. tall, 15 May 1978, *S. Nazimuddin* & *S. Abedin 680* (***Holotype***: KUH!). Illustrated by Ali (1983: 36).

Herbs to undershrubs, up to 60 (–100) cm tall. Stem longitudinally striate, pubescent along two longitudinal lines, sometimes moderately pubescent all around, internodes 1–7 cm long. Leaves opposite, decussate, lower and upper leaves smaller than the middle ones, petiolate; petioles 2–8 mm long, glabrous to sparsely pubescent, sometimes pubescent along the adaxial channel only; lamina mostly narrowly ovate to narrowly or broadly lanceolate, rarely elliptic-ovate, sparsely pubescent on both sides, 3–6 × 1–2 cm; apex acute; base mostly obtuse to sometime sub-acute; margins smooth, mostly pubescent; secondary veins 7–9 on each side of midvein, visible on both surfaces, sometimes sunken, pubescent. Inflorescences axillary, mostly sessile, often both sessile and short- to long-pedunculate inflorescences present on the same plant; peduncles up to 3 cm; bracts narrow, ca. 1 mm long, pubescent; pedicels 2–4 mm long, sparsely or densely pubescent. Flowers bicolored: calyx lobes green, and lower half of corolla lobes purple, upper half green, corona and gynostegium purple; sepals ovate or tapering into acute to narrowly acute apices, ca. 1 mm long, narrowly ovate, ca. 0.5 mm long, a few marginal and surface trichomes present, calycine colleters 5 per flower; corolla tube ca. 1 mm long, lobes oblong-ovate, ca. 2 × 1 mm, pubescent within, apex obtuse or occasionally emarginate; corona lobes subulate, ca. 1 mm long, less than 0.5 mm broad, much longer than gynostegium, apices approaching those of the opposite corona lobes, convergent; staminal appendages obtuse. Follicles narrowly ovate to fusiform, 5–9.5 × 1–1.5 cm, glabrous, inconspicuously striate, apex acuminate. Seeds 6–9 × 4–6.5 mm, dorsally dotted, lateral wings less than 1 mm broad; coma white, ca. 1.5 cm long.

##### Distribution and habitat.

Endemic to Zhob and Qila Saifullah districts in northeast Balochistan, Pakistan. It is found on dry stream beds made up of soil, stones and gravel. Its elevation ranges from 1300 to 2100 m.

##### Phenology.

*Vincetoxicum stocksii* flowers from April to June and fruits from June to August.

##### Provisional conservation status.

Vulnerable (Table 1). *Vincetoxicum stocksii* is endemic to two districts in Balochistan province, Pakistan. The populations are partially fragmented and face the threat of habitat loss especially on the dry stream beds due to rains.

##### Notes.

*Vincetoxicum stocksii* is a member of the purple-flowered group. Before its description as a new species by Ali and Khatoon (1982), it was identified as *V. arnottianum*. Recently, Shah et al. (2018) disintegrated *V. stocksii* into two species, separating from it *V. luridum*, an endemic of southwest Balochistan based on indumentum type on vegetative parts, corona lobes shape, pollinarium morphology and seed coat ornamentation. *Vincetoxicum stocksii* can be easily differentiated from *V. arnottianum* by the smaller pubescent leaves, the non-twisted and internally pilose corolla lobes, and the subulate corona lobes. These characters also differentiate it from *V. sakesarense*. The presence of both sessile and distinctly pedunculate inflorescences on the same plant is a common character shared between *V. stocksii* and *V. lenifolium*. However, green flowers, glabrous corolla lobes and smaller rhomboid corona lobes readily distinguish *V. lenifolium* from *V. stocksii*.

##### Specimens examined.

**Pakistan. Balochistan**: Northeast Balochistan: ca. 30 km from Zhob on way to Quetta, 07 July 1988, *T. Ali & T. Ahmad 23361* (KUH); Janabad: ca. 33 km from Zhob on way to Qila Saifullah, 1 June 1995, *T. Ali & G.R. Sarwar 2754* (KUH); ca. 20 km from Qilla Saifullah on way to Zhob (Fort Sandeman), 19 May 1984, *S. Omer & A. Ghafoor 1640* (KUH); Murgha (Balochistan), 25 October 1950, *A.H. Khan* s.n. (RAW); Lakkaband, 21 May 1896, *J.F. Duthie* s.n. (RAW); Hindubagh, 7000 ft., 24 October 1969, *Shariq 8318 & 8322* (PFI); Quetta, in valle 12 km. N. Murgha Kibsai, 30°48'N, 69°25'E, substr. Tonschiefer, 1600 m, s.d., *K.H. Rechinger 29803* (K); Quetta: Murgha Kibsai to Fort Sandeman, ca. 30 km from Fort Sandeman, stony and sandy plain, ca. 1500 m, 19 May 1965, *J. Lamond 1440* (MO); Zhob: 15 km towards Quetta, 1390 m, 17 August 2016, *R. Khan, Z. Abedin, N. Khan, B. Gul SAS-21* (RAW).

**Figure 15.**
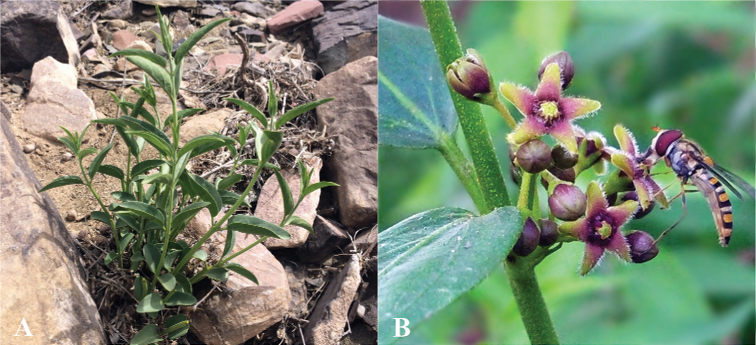
Field and garden photographs of *Vincetoxicum stocksii***A** habit **B** flowers. **Note**: A potted plant was photographed at the Botanical Conservatory, NARC; Islamabad. The insect is a honey bee. We did not observe successful pollination in the botanical conservatory in three years. Photos by S.A. Shah.

**Figure 16.**
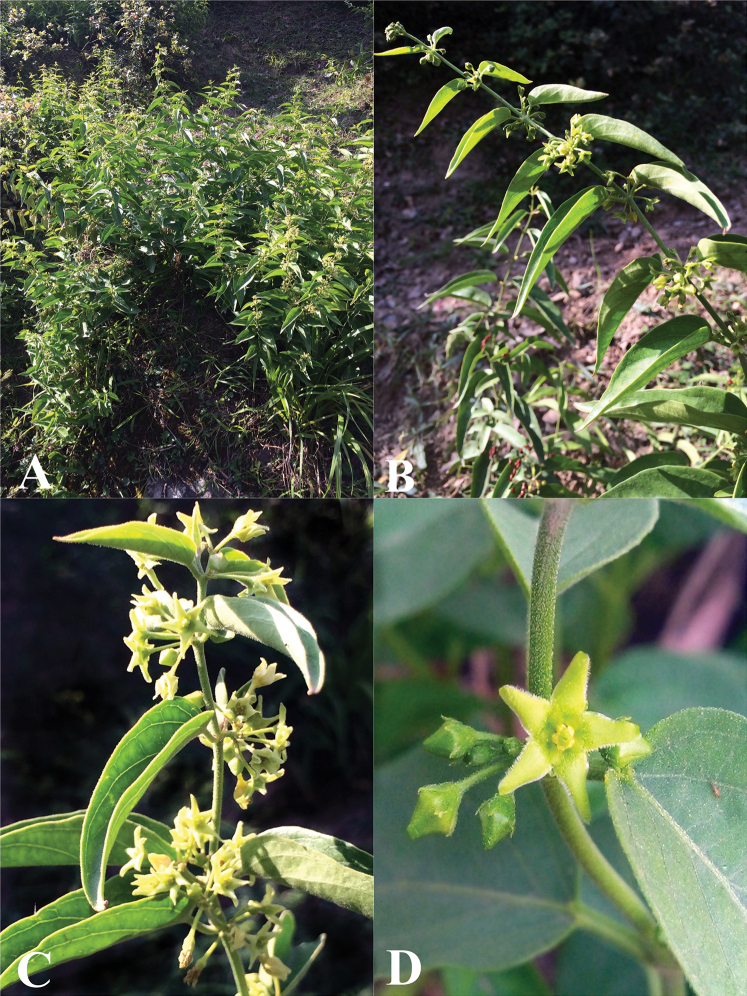
Field photographs of *Vincetoxicum subcanescens***A** habit **B, C** leaves and inflorescences **D** flowers. Photos by S.A. Shah.

#### 11. Vincetoxicum subcanescens

Taxon classificationPlantaeGentianalesApocynaceae

S.A. Shah & A. Sultan sp. nov.

24DCF637-3DD4-5028-A2BD-B62B91E64738

urn:lsid:ipni.org:names:77217790-1

##### Diagnosis.

Differing from *V. cabulicum* by having distinctly petiolate leaves, green flowers, and clavate corona lobes. In *V. cabulicum*, leaves are sessile, flowers dark purple, and corona lobes obovate.

**Figure 17.**
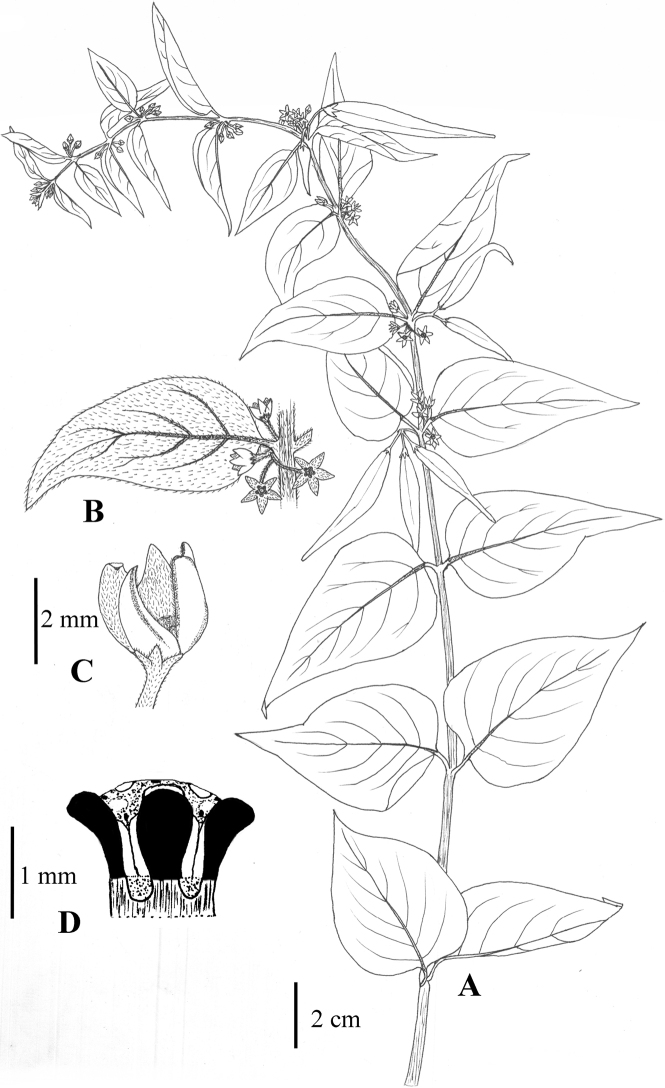
*Vincetoxicum subcanescens* illustrated by S.A. Shah & S.U. Nisa from *S.A. Shah & A. Ullah SAS-8* (RAW!) **A** plant with flowers and fruits **B** flowering node and leaf **C** flower **D** corona (black).

##### Type.

Pakistan. Dir Upper, Patraak, mountain slope near river, 1600 m, 16 June 2015, *S.A. Shah & A. Ullah SAS-8A* (***Holotype***: RAW!).

Undershrubs, up to 75 cm tall. Stem and other vegetative parts sub-canescent, internodes 1–9 cm long, striate. Leaves opposite, rarely whorled on some nodes; petioles 1–18 mm long; lamina discolorous, narrow to broadly ovate to sometimes lanceolate-ovate, 3–9 × 1.2–4.8 cm; apex acute to narrowly acute to shortly acuminate; base rounded to sub-cordate to sub-truncate; veins visible on both sides, more prominent on adaxial side, secondary veins 6–12 on each side of midvein, tertiary veins sometimes visible, lamina sub-canescent on both sides. Inflorescences sessile or both sessile and pedunculate inflorescences present on the same plant; peduncles up to 2.5 cm long, sub-canescent; bracts narrow with a few marginal trichomes; pedicels 2–7 mm long, pubescent. Flowers green to yellowish-green, 3–4 × 2–2.5 mm; sepals tapering into acute to narrowly acute apices, pubescent along margins and abaxial surface, up to 2 mm long, calycine colleters single or in pairs; corolla tube ca. 1 mm long, lobes tapering into obtuse apex, 2–2.5 × 1–1.5 mm, margins appearing wavy in dry flowers, sub-bearded within; corona lobes clavate, 0.5–1 × 0.2–0.5 mm, reaching the base, the middle or, rarely, the apex of the staminal appendages, divergent. Follicles fusiform, 4.5–9.5 × 0.7–1 cm, sparsely pubescent, minutely striate, apex narrowly acuminate. Seeds 5–6 × 2–3 mm, wings up to 1 mm broad; coma up to 3 mm long.

##### Distribution and habitat.

Endemic to Pakistan (Chitral, Dir, Swat and Gilgit Baltistan), Kashmir (Ladakh) and China (Tibet) . The habitat of the species is open slopes. Soil type is clay to gravel to large size stones. The elevation ranges from 1600 to 2800 m.

##### Etymology.

The name is based on the sub-canescent indumentum on the vegetative parts of the plant.

##### Phenology.

*Vincetoxicum subcanescens* flowers from April to August and fruits from May to October.

##### Provisional conservation status.

Least concern (Table 1). *Vincetoxicum subcanescens* is common in its range. However, the populations are extremely distant and clumped.

##### Vernacular name.

Lovaki (in Chitrali language).

##### Notes.

*Vincetoxicum subcanescens* was previously misidentified as *V. canescens* (Ali and Khatoon 1982; Boissier 1879). Rechinger (1970) treated it as *V. glaucum* in Flora Iranica and also synonymized *V. cabulicum* with it. *Vincetoxicum subcanescens* differs from *V. canescens* by the subcanescent indumentum, smaller flowers, clavate corona lobes, and glabrous and narrowly fusiform follicles. *Vincetoxicum canescens* is distributed in the eastern Mediterranean region in Turkey, Greece, Syria and Iraq. It is characterized by canescent indumentum, larger flowers, arrow-shaped corona lobes, and ovate and canescent follicles. *Vincetoxicum glaucum* exhibits variable leaf shapes, sub-glabrous or glabrous leaf surfaces, sessile inflorescences and toothed corona lobes. It is distributed in the Himalayas of India and Nepal. The closest relative of *V. subcanescens* is *V. cabulicum* which differs by the sub-sessile leaves and dark purple corolla and is endemic to northern Afghanistan.

##### Specimens examined.

**China. Tibet**: Hab. Tibet, 10,000–12,000 ft., s.d., *J. J. s. n.* (GOET [GOET020098; GOET020099]).

**Kashmir.** Nurla, Ladak, Kashmir. On sandy slope, 28 August 1931, *W. Koelz 2707* (RAW, US); North of Kamri Pass, above Shankargarh, ca. 10,000 ft., s.d., *R.R. Stewart 22773* (K, US); Kargil, Ladakh, Kashmir, 27–28 July 1933, *W. Koelz 6132* (US).

**Pakistan. Khyber Pakhtunkhwa**: Chitral, Rosh Gol Tirich Mulkhow, 9039 ft., dry hard soil, 08 October 2013, *Kifayat 516* (HUP); Chitral to Pirpesh, 8 June 1958, *I.I. Choudhri 7* (RAW); Dir [Upper]: Patraak, 13 July 1968, *Y. Nasir 5089* (RAW); Swat: Batain above Ushu cliffs, 27 July 1953, *R.R. Stewart & A. Rehman 25301* (RAW); **Gilgit Baltistan**: Satpura Nullah near Skardu, s.d., *A. Ghafoor 567* (KUH); Near Rattu above Astor, 21 August 1939, *R.R & I.D. Stewart 18834* (RAW); Bagocha to Olding, Indus valley, ca. 8500 ft., 22 August 1940, *R.R. Stewart 21009* (RAW); Indus Valley: below Parkutta, 20 August 1940, *R.R. Stewart 20908* (RAW); Between Arkote and Biafo base camp, 3200 m, 05 June 1962, *Schussalide 1095* (KUH); Astor: ± 30 km from Pagru on way to Shimshol, ±3000 m, 25 June 2007, *J. Alam et al. 3941* (KUH); Astor: Gorikot P.R.C, 26 May 2008, *A. Noor & Basharat 1489* (KUH); Astor: Peer rant village, bank of cultivated field, 12 September 2004, *A. Noor 52* (KUH); Astor: above Rattu, 2642 m, 23 August 2014, *A. Sultan SAS-52* (RAW); Hunza, Gilgit, ±35 km from Posu on way to Shimshol, 28 June 2007, *J. Alam, Karimuddin & M. Khan 3941* (KUH); Dharkot, 10,500 ft., 19 June 1976, *B. Lyon 8144* (KUH); Skardo-Dras, 8800 ft., petals green, acute, s.d., *C.B. Clarke 30512A* (K); B-9 Baltistan, Paskyum, 9600 ft., flowers greenish, s.d., *B.B. Osmaston 129* (K); Gilgit: Chamrot, 29 July 1957, *M.B. Zaman & D.K. D 1876* (PFI); Gilgit: Passu between lake and glacier, 16 August 1994, S.Z. *Hussain, R.A.W. Lowe, M. Shah & L.S. Springate 632* (PMNH).

## Supplementary Material

XML Treatment for Vincetoxicum arnottianum

XML Treatment for Vincetoxicum cabulicum

XML Treatment for Vincetoxicum cardiostephanum

XML Treatment for Vincetoxicum glaucum

XML Treatment for Vincetoxicum kenouriense

XML Treatment for Vincetoxicum lenifolium

XML Treatment for Vincetoxicum luridum

XML Treatment for Vincetoxicum sakesarense

XML Treatment for Vincetoxicum stewartianum

XML Treatment for Vincetoxicum stocksii

XML Treatment for Vincetoxicum subcanescens
